# The Use of an Ultrasonic Field in Support of Classical Methods of Oxidising Component Leached from Microplastics in Bottom Sediments

**DOI:** 10.3390/ma14113029

**Published:** 2021-06-02

**Authors:** Małgorzata Kida, Sabina Ziembowicz, Piotr Koszelnik

**Affiliations:** Department of Chemistry and Environmental Engineering, Faculty of Civil and Environmental Engineering and Architecture, Rzeszów University of Technology, AVE Powstańców Warszawy 6, 35-959 Rzeszów, Poland; s.ksiazek@prz.edu.pl

**Keywords:** di(2-ethylhexyl) phthalate, Fenton process, ultrasounds, microplastics, hazardous materials

## Abstract

The work detailed here examined the impact of selected unit methods and ultrasonic removal of the widespread plastic additive di(2-ethylhexyl) phthalate (DEHP) from the bottom sediments of a body of water. To this end, hydrogen peroxide and a classic or modified Fenton process were used, supplemented by an ultrasonic field. The latter had a vibration frequency of 20 kHz and an acoustic wave intensity of 3.97 W/cm^2^. The impact of process parameters such as reaction environment, reaction time, initial impurity content, aging of the impurity, influence of processes on the content of organic matter and dissolved organic carbon, and elution of selected components from the matrix were all analysed. It emerged that the most effective process by which to remove DEHP from a solid matrix involved a modified Fenton process assisted by an ultrasonic field. The highest average degradation efficiency achieved in this way was 70.74%, for *C*_0_ = 10 mg/kg d.w. and *t* = 60 min.

## 1. Introduction

Plastics began to be produced on a large scale in all branches of the economy in the late 1940s. Scientists estimate that about 9.1bn tons of the material have been generated since. Many factors, including light, heat, water and oxidation are responsible for plastic’s degradation in the aquatic environment, but the process facilitates the transfer of many toxic chemicals into the aquatic ecosystem as plastics are obtained by combining polymers with such additives as fillers, stabilisers, plasticisers, dyes, pigments, antistatic agents, antioxidants, flame retardants and other substances that improve polymer properties. Plasticisers in particular can readily migrate out of a plastic element, having not reacted chemically with it, but only having formed physical bonds [1,2,3,4,5].

The plasticisers used most commonly include the phthalic acid esters (PAEs) whose global annual production is an estimated 2.7 million tons. Within that total, some 25% is accounted for by di(2-ethylhexyl) phthalate (DEHP) (Supplementary Materials, Table S1) [4,6,7]. These are usually stable compounds in the environment, showing lyophilic properties, and having a tendency to bioaccumulate [6,8,9]. Furthermore, the high environmental durability of the polymer matrix ensures years of potential release of phthalates from finished products. Indeed, an assumed annual emission of 1% denotes a half-life for DEHP of 69 years, and complete degradation only after more than a century [6,10]. Due to low solubility in water, Koc value and Henry’s law constant (Supplementary Materials, Table S2), the highest content of di(2-ethylhexyl) phthalate is that to be found in bottom sediments of waters (maximally 322 mg/kg d.w. in bottom sediments of China’s Yangtze River) [10]. Overall, though, DEHP is to be noted in air, precipitation, drinking water, groundwater, surface water, the soil, bottom sediments and living organisms (Supplementary Materials, Tables S3–S6) [11].

Products that only decompose with difficulty may nevertheless be removed to a high level of efficiency using chemical methods. From among the Advanced Oxidation Processes (AOPs), it is the Fenton process whose use is recommended in the remediation of soil and bottom sediments [12,13]. However, in its classical form, this process is characterised by kinetics that are too slow to allow for the degradation of pollutants highly-absorbed in soils and bottom sediments, and the situation is rendered all the more problematic, given the heterogeneous nature of these matrices [14,15,16]. Ways of modifying the Fenton process for the purposes of soil remediation are primarily a reflection of a lower availability of impurities that reflects sorption on solid particles, as well as hindered operation of the iron catalyst in a natural soil environment. In the face of this, a promising method entails deployment of an ultrasonic field (UD) in support of the Fenton process, given the simultaneous obtainment of pollutant desorption and pollutant decomposition. The combined processes inter alia allow for markedly increased efficiency of removal of impurities as compared with the unit process, with time needed often reduced, along with reduced dose of oxidiser or catalyst, depending on the processes used.

Due to the lack of effective methods for removing plasticizers from the water environment, the study analyzes the impact of single processes and their support with an ultrasonic field on the effectiveness of removing substances from the group of phthalic acid esters (plasticizers). There is still a need for complex laboratory scale research due to the lack of data on this subject. Advanced oxidation methods require well-defined reaction conditions that must be determined at the laboratory stage. Selecting process parameters appropriate to a specific substance or group of substances, or else the correct combination with other unit processes, requires detailed recognition, and further study of the mechanism involved. In line with this knowledge and with the additional aim of the use of ultrasonic waves promoted as a clean environmental protection technology, preliminary research has sought to indicate the factors on which the degree of DEHP degradation depends, in line with the application of the tested processes.

## 2. Materials and Methods

### 2.1. Chemicals

The di(2-ethylhexyl) phthalate (DEHP) and di(2-ethylhexyl) phthalate-3,4,5,6-d4 (DEHP-3,4,5,6-d4) used in trials were supplied by Sigma–Aldrich (Darmstadt, Germany). Hydrogen peroxide solution (30%), FeSO_4_·7H_2_O, Fe(NO_3_)_3_·9H_2_O, Na_2_SO_4_, NaOH, HCl were in turn all obtained from Chempur (Piekary Śląskie, Poland). The cellulose acetate membrane filters of 0.20 and 0.45 µm pore size and the syringe filter of 0.22 µm pore size were purchased from LaboPlus (Warsaw, Poland), while analytical-grade n-hexane, methanol and acetone were obtained from POCH (Gliwice, Poland). Ultra-pure water was obtained from Purix CNX-100, Krakow, Poland). All glassware was submerged in NaOH solution for 24 h, dried for 5 h and then rinsed with acetone prior to heating at 280 °C for 5 h.

### 2.2. The Samples of Bottom Sediment

Uncontaminated bottom sediments were collected from a reservoir located in Rzeszow (SE Poland). A gravity sediment corer (KC Kajak of Denmark, Germany) was used in all cases. The sediments were stored in glass amber jars, which had been pre-washed with acetone. They were passed through a 1.0 mm sieve, pre-washed with acetone (3 × 24 h) and dried to constant weight (105 °C). These dried sediments were homogenised and stored at 4 °C. Real bottom sediments were found to contain 8.45% organic matter (OM) and 4.38 mg/g d.w. (dry weight) of dissolved organic carbon (DOC), while those washed with acetone had corresponding figures of 7.78% OM and 4.01 mg/g d.w. DOC. pH values were 7.88 for the real bottom sediments and 7.95 in the case of the washed sediments.

1 g samples of cleaned bottom sediments were placed in glass reaction vessels supplied with acetone and DEHP in appropriate amounts (in the range 10–100 mg/kg d.w.). Samples were mixed to disperse DEHP for 1 h at 150 rpm in closed vessels without light. The solvent was evaporated for 24h under a hood. To achieve a process of sequestration of pollution, bottom sediments containing DEHP (at 50 mg/kg d.w.) had been stored at room temperature without light for 6 months.

### 2.3. Experimental Conditions for the Degradation

Unit processes such as the classic (H_2_O_2_/Fe^2+^) and modified Fenton process (H_2_O_2_/Fe^3+^), or a process using hydrogen peroxide (H_2_O_2_), were carried out in a volume of 3 mL (1:3 *w*/*v*) aqueous phase. In contrast, in line with the technical requirements for the apparatus, processes supported by the ultrasound field were tested on a volume of 40 mL of aqueous phase. The conducted preliminary studies aiming at the correct selection of the amount of the water phase showed that the unit processes (H_2_O_2_, H_2_O_2_/Fe^2+^, H_2_O_2_/Fe^3+^) require a smaller amount of the water phase than the processes supported by the ultrasonic field. Unit processes were carried out in a volume of 40 mL, but no removal of DEHP was recorded at this volume of the aqueous phase. This was probably due to the low availability of hydroxyl radicals to the contamination. Detailed research in this area is presented in the article by Kida et al. (2020) [17]. The analyzed parameters and factors in the tested processes are summarized in Table 1.

A SONOPULS HD 3200 source of ultrasound—from Bandelin (Berlin, Germany)—was used, the frequency being 20 kHz and the nominal power 200 W. The device is equipped with a titanium probe tip of 13 mm diameter. Maximum vibration amplitude up to this tip is 170 μm. The sono-chemical reactor was thermostated using the water jacket. The temperature of the sediment water suspension was 20 ± 1 °C, given the possibility of volatilisation of DEHP from the sample or/and thermal degradation. The sonication probe was dipped 1cm below the sediments’ water suspension surface. 1 g of sediment was sonicated for 5, 15, 30 and 60 min. The tests were conducted in an open-air system, used to check the effectiveness of DEHP removal without additional costs being incurred.

In the process using hydrogen peroxide, or else in the classical and modified Fenton process, amounts of reagents were determined in molar–ratio terms. After the aqueous phase had been introduced and the pH value stabilised, the catalyst was introduced into the system first (except in the case of the process using hydrogen peroxide only). The samples were mixed for 10 min to distribute the catalyst evenly. Reactions were initiated by the gradual introduction of an oxidant (hydrogen peroxide). The slurry was stirred vigorously at 250 rpm during the process. In the processes supported by the ultrasonic field, 40 mL of distilled water was introduced into 1 g of contaminated bottom sediments. Then, the pH was stabilized to the required value. Then, an appropriate amount of the catalyst was introduced into the system (in appropriate processes) and mixed to obtain the homogeneity of the reaction mixture. The sample was subjected to an acoustic wave, during which hydrogen peroxide was gradually introduced. The subsequent stages of the test procedure were analogous to the earlier methods used.

The reaction was terminated by introducing 1M NaOH or 1M H_2_SO_4_ into the reaction solution, to adjust the pH to neutral. All the experiments were done in triplicate, with an observed deviation of less than 5%. For this reason, average values are shown in the graphs. All tests were also conducted at room temperature and pressure.

### 2.4. Chemical Analysis

Extraction of DEHP from bottom sediments was carried out using microwave radiation with a MARS 6 mineraliser/extractor (SELWALab, Warsaw, Poland) in the presence of 10 mL methanol. The applied device’s operating temperature—120 °C, max power—1400 W, time to rise to temperature—20 min, heating time—10 min. Extraction was carried out in the presence of the DEHP-3,4,5,6-d4 internal standard. The drying step was carried out by adding a drying agent (anhydrous Na_2_SO_4_) which was activated by calcining in a muffle furnace at 550 °C for 4 h. The obtained solid-phase extracts were concentrated to a volume of 1 mL under a stream of nitrogen. The obtained extracts were then subjected to chromatographic analysis using a gas chromatograph coupled with a GC/MS mass spectrometer (Thermo Scientific, Waltham, MA, USA). Separation was achieved along a 30 m × 0.25 mm I.D. DB-5MS column (Thermo Scientific, Waltham, MA, USA) coated with 5% phenyl-95% dimethylpolysiloxane (film thickness 0.25 mm). Temperature program: 90 °C to 280 °C; heating rate: 20 °C/min; temp. max. holds for 10 min. MS conditions: transfer line 280 °C, ion source 210 °C. Helium was the carrier gas (flow: 1.2 mL/min). Internal-standard quantification was applied, and the method proved to be linear, with regression coefficient (R^2^) > 0.99. In the identification of by-products, the spectral library search method was used to interpret the mass-spectra results, by reference to retention times, and the characteristic fragmentation ions of the substances analysed.

The organic matter (OM) content in bottom sediments was determined by the weight method, entailing calcining of the sample in a muffle furnace for 5 h at 550 °C. The content of dissolved organic carbon (DOC) was in turn determined using a TOC analyser (Shimadzu, TOC-V CPN, Kyoto, Japan). Bottom-sediment extracts were centrifuged for 10 min at 8000 rpm thereafter, then filtered through a cellulose acetate membrane filter of pore size 0.45 μm. Samples were fixed by adding concentrated sulphuric acid to obtain a pH ≤ 2. Until the measurements were taken, the samples were stored at <4 °C. The pH of the bottom sediment was determined using the potentiometric method. Freshly-prepared distilled water was poured into 1 g of air-dried sediments in a ratio of 1:3 (*w*/*v*). The suspension was mixed intensively (5 min) with a glass rod, after which the mixture was left to stand for 24 h. The pH was measured using a HACH HQ30d portable single-channel multichannel meter.

Analysis further extended to the leaching of ingredients, including the most important plant nutrients, from bottom sediments after the processes analysed had been run. Resulting aqueous extracts for the determination of calcium, magnesium and potassium were filtered through 0.22 μm syringe filters and purified on OnGuard II filters (Thermo Scientific, Waltham, MA, USA). The content of these components was analysed using a DIONEX DC ICS-5000 ion chromatograph (Thermo Scientific, Waltham, MA, USA). An ion chromatograph equipped with a conductometric detector, an automatic sample feeder and a 4 mm CSRS ion suppressor were used. Column: Dionex CS 17 250 mm × 4.0 mm (Thermo Scientific, Waltham, MA, USA). Mobile phase: 6 mM methane-sulfonic acid in water. Mobile phase flow: 1 mL/min. In turn, for determinations of Ni, Pb, Al, Cu and Zn, HNO_3_ at an amount of 5 mL was introduced into the obtained aqueous extracts at a volume of 20 mL and made up to 50 mL with distilled water. Aqueous extracts obtained passed through a membrane filter of pore size 0.20 μm, and were analysed using a GBC Quantima E 1330 ICP-OES (Braeside, Australia).

### 2.5. Statistical Analysis

Results were analysed with MS Excel 2013 and Statistica 13. Non-parametric statistical analyses were carried out, given the uneven variances and sample sizes characterising the conducted analyses. Homogeneity of variance was checked using Brown-Forsythe and the Levene Tests. Hypothesis testing used the criterion of differences significant at α ≤ 0.05. To assess data structure, and the relationship between the variable and factors contributing to DEHP degradation, a principal component analysis (PCA) was performed.

## 3. Results and Discussion

### 3.1. Determining the Optimal Dose of Reagents and pH of the Reaction Medium

#### 3.1.1. The Process Using Hydrogen Peroxide (H_2_O_2_) and Its Support with an Ultrasonic Field (H_2_O_2_/UD)

**Figure 1:**
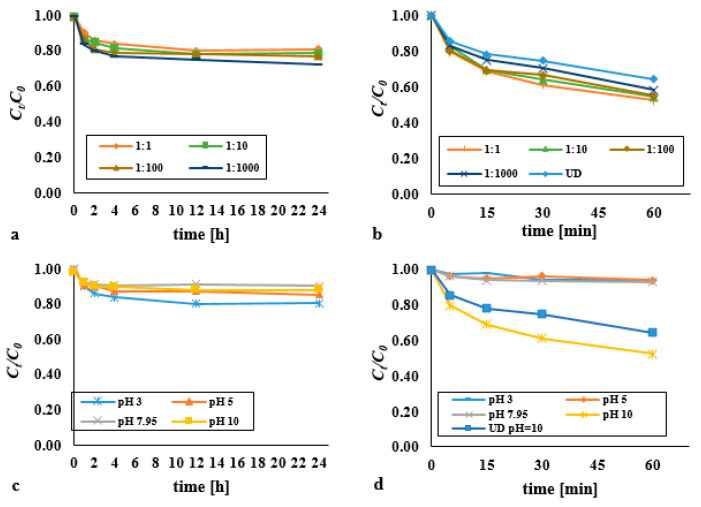
Impact on the decomposition of DEHP exerted using (**a**) hydrogen peroxide in various doses (pH = 3); (**b**) hydrogen peroxide in various doses supported by an ultrasonic field (pH = 10, acoustic wave intensity - 3.97 W/cm^2^); (**c**) hydrogen peroxide in various pH (molar ratio DEHP:H_2_O_2_ 1:1); (**d**) hydrogen peroxide supported by an ultrasonic field in various pH (molar ratio DEHP:H_2_O_2_ 1:1, acoustic wave intensity—3.97 W/cm^2^).

The efficiency of removal of DEHP from bottom sediments using hydrogen peroxide as facilitated by means of an ultrasonic field was analysed for DEHP:H_2_O_2_ molar ratios of 1:1, 1:10, 1:100, and 1:1000 at constant DEHP content equal to 0.13 mM/kg d.w. (Figure 1). The effectiveness of DEHP degradation was as presented in the form of the *C_t_*/*C*_0_ = f(*t*) relationship (*C_t_*—the DEHP content in bottom sediments after time *t*, *C*_0_—the initial content of DEHP in bottom sediments). Single processes were carried out over a longer time due to the slower course of the reaction; the reaction time was *t* = 24 h. In the first hour of the process, no significant differences in the efficiency of DEHP removal were noted. Therefore, the duration of the process was extended to 24 h. On the other hand, conducting research supported with the ultrasound field exceeding 1 h is economically ineffective.

No significant differences in the efficiency of DEHP removal using hydrogen peroxide were observed across the range of DEHP:H_2_O_2_ molar ratios from 1:1 to 1:1000 (Figure 1a). However, a statistically significant difference was found in the DEHP content over time for the dose 1:1 (*p* < α, *p* = 0.0194), 1:10 (*p* < α, *p* = 0.0217), 1:100 (*p* < α, *p* = 0.0319), 1:1000 (*p* < α, *p* = 0.0144). Irrespective, after 24 h, the degree of degradation had not exceeded 30%. Where the dose of hydrogen peroxide was still higher, the results were not as expected, though the reaction proceeded more efficiently at lower pH values (Figure 1c). The most effective oxidation of organic substances with H_2_O_2_ usually takes place under alkaline conditions, with this reflecting the formation of oxidising HO^2−^ (hydrogen-peroxide(1-)), and indirectly HO· (hydroxyl radical) and HO_2_· (hydro-peroxy radicals). The higher efficiency of DEHP removal in an acidic environment compared to alkaline may result from access to iron ions contained in the analysed sediments (34.58 g/kg d.w.). In their research, Ledakowicz et al. (2001) [18], after 26 h using H_2_O_2_ (0.15 mM/L), obtained a reduction of the Triton X-114 (100 mg/L) nonionic surfactant at a level of just 4%. The low efficiency was due to the low level of decomposition of H_2_O_2_, therefore giving rise to only negligible amounts of hydroxyl radicals in solution.

The introduction of the 3.97 W/cm^2^ acoustic wave into the system resulted in a shorter response time (*t* = 5–60 min) and a significant increase in the efficiency of DEHP removal from bottom sediments (Figure 1b). Sono-chemical and sono-physical reactions significantly accelerated the degradation of DEHP compared to their unsupported counterparts (Supplementary Materials, reactions 1–13). However, an acidic reaction medium was more favourable to the removal of di(2-ethylhexyl) phthalate from bottom sediments where oxidation with hydrogen peroxide was tested, while basic conditions were more favourable in the case of sonification (Figure 1d). Given the pKa value, the substance in question should undergo sono-chemical degradation across the entire range of pH values. However, as earlier studies [17] showed, low pH values encourage elution of hydroxyl-radical scavengers from the matrix, with the result that decomposition of DEHP is inhibited. The presence of humic compounds, complexing compounds, HCO_3_¯, CO_3_¯, phosphate and bromide ions, formaldehyde, tert-butyl alcohol, citric acid, oxalic acid, formic acid and acetic acid, and many others, may inhibit or intensify of the pollutant removal. They undergo oxidation, acting as the scavenger of highly reactive hydroxyl radicals.

Where the reaction medium in the combined process was acidic, the consequence was inhibited degradation of di(2-ethylhexyl) phthalate. In turn, after 60 min an alkaline (pH 10) reaction combined with the ultrasound-assisted process a rate of degradation higher by 41.7% was observed in comparison with the process entailing hydrogen peroxide as the oxidant. Equally, the ultrasound field applied on its own was found to be 11.17% less effective (at *t* = 60 min).

A statistically significant difference was found in the DEHP content over time for the dose 1:1 (*p* < α, *p* = 0.0156), 1:10 (*p* < α, *p* = 0.0156), 1:100 (*p* < α, *p* = 0.0237), 1:1000 (*p* < α, *p* = 0.0156). Best results were obtained where the acoustic wave was applied to a mixture with a DEHP:H_2_O_2_ molar ration equal to 1:1. The process was actually inhibited in the remaining cases studied. Where the concentration of hydrogen peroxide is too high, the main fate of hydroxyl radicals is to be consumed by further reaction with H_2_O_2_ (H_2_O_2_ + HO· → HO_2_· + H_2_O), with amounts of HO· reduced in consequence, along with overall process efficiency [19,20].

Despite the fact that hydrogen peroxide is described as an ecological and strong oxidant, when used individually, it turned out to be too weak for effective degradation of di(2-ethylhexyl) phthalate. Moreover, lowering the pH value is connected with the necessity of subsequent alkalisation of the bottom sediments. Consequently, new solutions are sought, e.g., by combining single processes and/or changing the conditions of the processes at higher pH values.

#### 3.1.2. The Fenton Process Unsupported (H_2_O_2_/Fe^2+^) or and Supported by an Ultrasonic Field (H_2_O_2_/Fe^2+^/UD)

**Figure 2:**
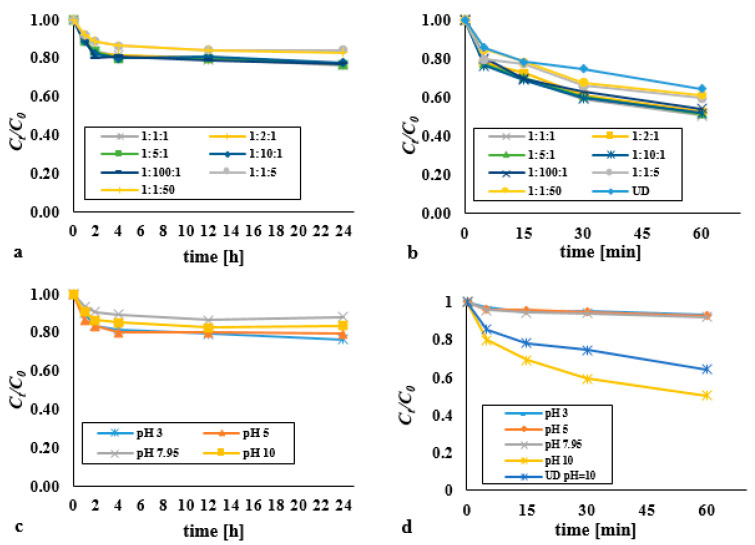
Impact exerted on the decomposition of DEHP where use is made of: (**a**) a Fenton process with various dosages (at pH 3); (**b**) a Fenton process with various dosages supported by an ultrasonic field (pH = 10, acoustic wave intensity = 3.97 W/cm^2^); (**c**) a Fenton process in various pH (molar ratio DEHP:H_2_O_2_:Fe^2+^ 1:1:1); (**d**) Fenton process assisted by ultrasonic field in various pH (molar ratio DEHP:H_2_O_2_:Fe^2+^ 1:1:1, acoustic wave intensity—3.97 W/cm^2^).

In the typical Fenton process and the process supported by an ultrasonic field, the influence of the DEHP:H_2_O_2_:Fe^2+^ molar ratio was examined by reference to 1:1:1, 1:2:1, 1:5:1, 1:10:1, 1:100:1, 1:1:5 and 1:1:50 variants, with a view to determining the relevance of this to the effectiveness of elimination of DEHP from bottom sediments (where the DEHP content was a constant 0.13 mM/kg d.w.) (Figure 2).

That said, it should always be recalled that the Fenton reaction is a complex process involving many side reactions (Supplementary Materials, reactions 14–35). Indeed, excess iron ions as well as excess hydrogen peroxide act as scavenger of hydroxyl radicals, with consequent inhibition of the process.

Nevertheless, across the analysed range of H_2_O_2_ doses, larger amounts of the oxidant relative to iron ions (to a 100:1 ratio) did not bring about any significant reduction in the efficiency of removal of DEHP (Figure 2a). Where economic considerations are concerned, the most effective dose therefore involves a DEHP:H_2_O_2_:Fe^2+^ molar ratio of 1:1:1. A statistically significant difference was found in the DEHP content over time for the dose 1:1:1 (*p* < α, *p* = 0.0091), 1:2:1 (*p* < α, *p* = 0.0174), 1:5:1 (*p* < α, *p* = 0.0114), 1:10:1 (*p* < α, *p* = 0.0186), 1:100:1 (*p* < α, *p* = 0.0310), 1:1:5 (*p* < α, *p* = 0.0118), 1:1:50 (*p* < α, *p* = 0.0102).

The only-slight difference in observed response when hydrogen peroxide, as opposed to the full Fenton process, was applied may reflect the naturally-occurring iron ions present in the bottom sediments tested, which may have been available as the process was ongoing. In soil and bottom sediments, the required H_2_O_2_:Fe^2+^ molar ratio should be achievable more readily than in aqueous solutions. Equally, the slight observed increase in the efficiency of removal of DEHP following the introduction of an additional amount of iron(II) ions into the reaction mixture reflects the formation of a surface complex between hydrogen peroxide and iron oxides present in the matrix. This step is followed by the transfer of an electron to the complex, with Fe^2+^ and HO_2_· generated. Excess hydrogen peroxide reacts heterogeneously with Fe^2+^, with the result that HO. forms on the surface of the solid matrix. The high reactivity of these radicals ensures that pollutants are only oxidised near their place of production. A share of ions in relation to hydrogen peroxide above 50% does not improve the effects of the process [21], but such determinations of optimal proportions between reagents is worthwhile, as undesirable radical reactions can be avoided [22]. Silva et al. (2009) [23] using the Fenton process to remove PAHs in soil, after 4 h achieved 94% efficiency in removing phenanthrene, and in the case of pyrene less than 50%. The effectiveness of the Fenton process is also largely determined by the nature of the oxidized organic compounds, their chemical structure, the polarity of molecules and the degree of aromaticity. Aromatic compounds are easily oxidized in the classic Fenton process, while aliphatic compounds require additional support from other processes [23].

The decomposition of DEHP was seen to be most effective at pH 3 (Figure 2c). In the Fenton process, the pH value should be in the 2.5–4 range, though below 3 the concentration of hydrogen ions is too high, ensuring that H^+^ ions are the main acceptors of hydroxyl radicals. A higher pH is also associated with a process of less efficiency, mainly because of the appearance of Fe(OH)_3_ (which does not react with hydrogen peroxide), as well as the lesser stability (faster degradation) of H_2_O_2_ [24,25,26]. This is also confirmed by studies by Esmaeli et al. (2011) [25], who using the Fenton process (H_2_O_2_−90 mg/L, Fe^2+^−5 mg/L) to remove DEHP (20 mg/L) from an acidic water solution (pH = 3) achieved 85.6% degree of degradation of this substance. Changing the pH to a value of 6 and also to a value of 2 resulted in a reduction in efficiency to approximately 35% and 45%, respectively. However, the Fenton process in alkaline bottom sediments was not inhibited, but was only less effective. For pH = 10, the average efficiency of di(2-ethylhexyl) phthalate removal at the level of 14.6% (*t* = 24 h), for pH = 3 at the level of 21.5% was noted. The bottom sediment could be a heterogeneous catalyst due to the content of iron and other ions, which exhibit catalytic activity. The main advantage of Fenton-like reactions is that they take place over a wide range (i.e., 2–10) of pH values. High pH values favor the formation of hydroxyl ions and, consequently, free hydroxyl radicals. At low pH values, H^+^ ions are formed and the ongoing reactions cause the formation of free hydrogen radicals [21,22,23].

The simultaneous application of the methods (H_2_O_2_/Fe^2+^/UD) results in a significantly higher efficiency of di(2-ethylhexyl) phthalate degradation where the reaction medium is basic (pH = 10) (Figure 2b,d). At the remaining values for pH of the reaction mixture it is inhibition of the process that ensues (as in the H_2_O_2_/UD system). There was no statistically significant difference in the DEHP content over time for pH 3 (*p* < α, *p* = 0.0586), 5 (*p* < α, *p* = 0.1718) and 7 (*p* < α, *p* = 0.0711). The acidic reaction medium of the sonication process was not suitable for the removal of DEHP from the bottom sediments. This may be the result of greater leaching of various substances from the analyzed bottom sediments. The presence of Ca^2+^ ions in the solution subjected to sonication may have a significant impact on the effectiveness of pollutant removal (Section 3.6). The high concentration of calcium inhibits the formation of hydroxyl radicals. The susceptibility of PAHs to decomposition in soil, sewage sludge and bottom sediments with the use of Fenton’s reagent was investigated by Flotron et al. (2005) [27]. They showed that the optimal process conditions depended on the type of matrix and its characteristics.

An excess of Fe^2+^ relative to H_2_O_2_ may also prove detrimental to (i.e., reduce the efficiency of) the process, probably because of reactions with the emerging HO· (Fe^2+^ + HO· → Fe^3+^ + OH^−^). However, the application of the ultrasonic field in the Fenton process was again associated with raised efficiency, depending on the dose used, where the comparison is with the classic Fenton’s reagent (at pH = 3), and the sonification process (pH = 10) (Figure 2b).

Results are thus clear in indicating a synergistic effect for advanced oxidation in the H_2_O_2_/Fe^2+^/UD system, for most analysed doses of reagents at least. For the dose of the reagents in the 1:1:1 molar ratio, the efficiency of DEHP removal in the Fenton process was 10% for pH = 3 and 7.3% for pH = 10 (*t* = 60 min), while for the ultrasound-assisted process the effectiveness increased to the value of 49.5% (pH = 10, *t* = 60 min). The use of the ultrasonic field resulted in the DEHP removal efficiency at the level of 36.4% (pH = 10, *t* = 60 min). A statistically significant difference was found in the DEHP content over time for the dose 1:1:1 (*p* < α, *p* = 0.0156), 1:2:1 (*p* < α, *p* = 0.0156), 1:5:1 (*p* < α, *p* = 0.0156), 1:10:1 (*p* < α, *p* = 0.0156), 1:100:1 (*p* < α, *p* = 0.0156), 1:1:5 (*p* < α, *p* = 0.0188), 1:1:50 (*p* < α, *p* = 0.0153). Optimum efficiency noted was with a 1:1 H_2_O_2_:Fe^2+^ molar ratio, to the extent that, just 5 min into the process, effects obtained were already comparable with those achievable with 24 h of the classic Fenton process (at pH 3).

#### 3.1.3. The Modified Fenton Process (H_2_O_2_/Fe^3+^) as Supported with an Ultrasonic Field (H_2_O_2_/Fe^3+^/UD)

**Figure 3:**
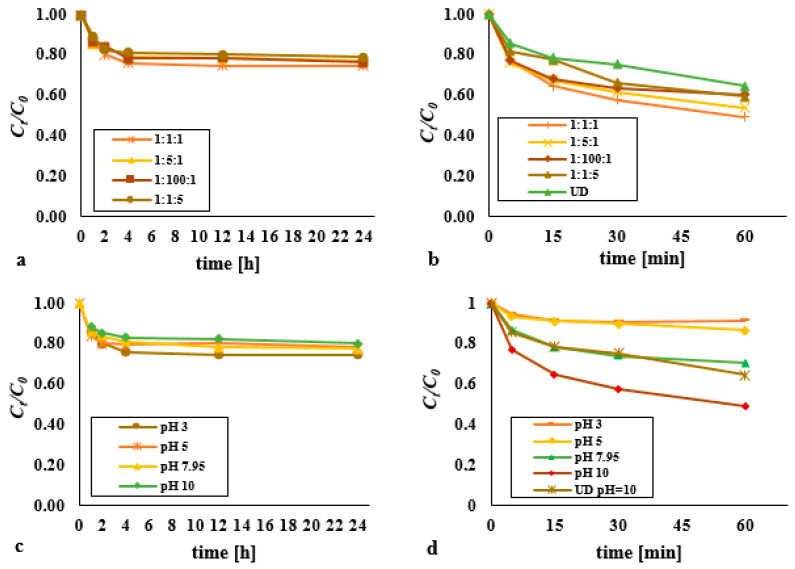
Impact on the decomposition of DEHP using (**a**) a modified Fenton process with various dosages (at pH = 3); (**b**) a modified Fenton process with various doses supported by an ultrasonic field (pH = 10, acoustic wave intensity of 3.97 W/cm^2^); (**c**) a modified Fenton process in various pH (molar ratio DEHP:H_2_O_2_:Fe^3+^ 1:1:1); (**d**) a modified Fenton process supported by an ultrasonic field in various pH (molar ratio DEHP:H_2_O_2_:Fe^3+^ 1:1:1, acoustic wave intensity of 3.97 W/cm^2^).

Modification of the Fenton process to use Fe^3+^ as the catalyst, rather than Fe^2+^, was first guided by the analysis of the classical Fenton process, in that doses of reagents were at the DEHP:H_2_O_2_:Fe^3+^ molar ratios of 1:1:1, 1:5:1, 1:100:1 and 1:1:5 (Figure 3a). A statistically significant difference was found in the DEHP content over time for the dose 1:1:1 (*p* < α, *p* = 0.0194), 1:5:1 (*p* < α, *p* = 0.0132), 1:100:1 (*p* < α, *p* = 0.0138), 1:1:5 (*p* < α, *p* = 0.0197). Under these circumstances, the maximum observed efficiency of removal of di(2-ethylhexyl) phthalate was 25.5% for *t* = 24 h, where the molar ratio was 1:1:1. That process was also most effective at pH 3 (Figure 3c). There was no statistically significant difference in the DEHP content over time for pH 5 (*p* < α, *p* = 0.1546). A statistically significant difference was found for pH 7 (*p* < α, *p* = 0.0244) and pH 10 (*p* < α, *p* = 0.0146).

An increase in the amount of H_2_O_2_ relative to iron (III) ions did not result in enhanced degradation of the pollutant, while excess Fe^3+^ was associated with a lower effectiveness by 21.1% on average. Comparison with the deployment of the classic Fenton’s reagent showed that removal of DEHP was at most 5.4% greater for *t* = 24 h. A factor here is most likely the presence of HO· and HO_2_, generated via the interaction between H_2_O_2_ and Fe^3+^:

Fe^3+^ + H_2_O_2_ → Fe^2+^ + HO_2_· + H^+^, and then: (1)

Fe^2+^ + H_2_O_2_ → Fe^3+^ + HO· + OH^−^. (2)

The use of Fe^3+^ ions denotes a limitation on the rate of reaction linked with the rate of formation of Fe^2+^. HO radicals are generated in a two-stage process, via a slow reaction between Fe^3+^ and H_2_O_2_, as followed by a rapid reaction between the generated Fe^2+^ and H_2_O_2_. Degradation of organic compounds is thus slower in the presence of Fe^3+^ than Fe^2+^, in a bottom-sediment matrix or soil, and this is advisable due to the need for earlier desorption of pollutants into the aqueous phase and rapid decomposition of hydrogen peroxide to oxygen and water [14,24,28].

In the analyzed pH range in the modified Fenton process, the best effects in the degradation of DEHP were obtained for pH = 3, and the removal efficiency was on average 25.5% for *t* = 24 h. The reaction was effective also for pH = 10, where the DEHP removal efficiency was recorded at 20%.

Results indicate limited DEHP removal where the medium was acid in reaction H_2_O_2_/Fe^3+^/UD (Figure 3b). Individual processes actually proved more effective than the combined system. However, in the natural reaction environment, strong support for the modified Fenton process did ensue when the ultrasonic field was applied. Furthermore, a synergistic effect was noted for the reaction mixture in which the pH value was 10 (Figure 3d). An increase in efficiency of 36.9% for pH = 3 and 39.1% for pH = 10 was observed compared with the modified Fenton process (UD—36.4%, H_2_O_2_/Fe^3+^/UD—51%, H_2_O_2_/Fe^3+^—11.9% for pH = 10, 14.1% for pH = 3, *t* = 60 min). The process supported by the acoustic wave (pH = 10) was most effective in the first 15 min, and in the following minutes a slight decrease in efficiency was observed, probably caused by leaching of hydroxyl radical scavengers from the bottom sediments to the water phase. Statistically significant differences were observed for the process supported by an ultrasonic field in time for the reagent dose 1:1:1 (*p* < α, *p* = 0.0156), 1:5:1 (*p* < α, *p* = 0.0156), 1:100:1 (*p* < α, *p* = 0.0232), 1:1:5 (*p* < α, *p* = 0.0156). In turn, Chiou et al. (2006) [29] analyzed the effect of supporting the H_2_O_2_/Fe^3+^ system with UV radiation on the removal of di-n-butyl phthalate (5 mg/L) from an aqueous solution. After 60 min of the reaction time, they achieved a 46% degree of DBP reduction as a result of the H_2_O_2_/Fe^3+^ process; the value of this parameter increased to 76.5% after the introduction of UV radiation with an intensity of 120 µW/cm^2^ into the H_2_O_2_/Fe^3+^ system.

### 3.2. Impact on Degradation of the Initial Content of Di(2-ethylhexyl) Phthalate

#### 3.2.1. The Process Using Hydrogen Peroxide as Supported by an Ultrasonic Field

**Figure 4:**
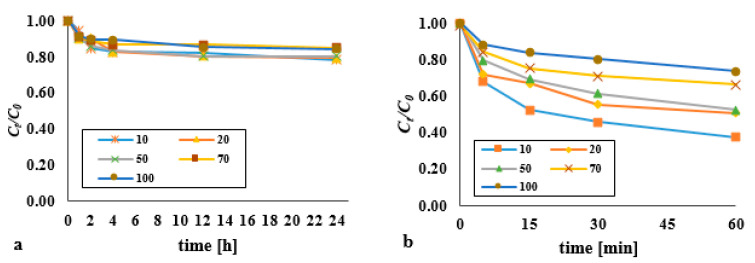
Influence of initial content on the decomposition of DEHP using (**a**) hydrogen peroxide (pH = 3, molar ratio DEHP:H_2_O_2_ 1:1); (**b**) hydrogen peroxide supported by an ultrasonic field (pH = 10, molar ratio DEHP:H_2_O_2_ 1:1, acoustic wave intensity of 3.97 W/cm^2^).

Successively higher initial DEHP *C*_0_ contents (of 10, 20, 50, 70 and 100 mg/kg d.w.) were associated with progressively lower efficiencies of removal of the substance (Figure 4). 1 h into the process of oxidation by hydrogen peroxide, the degree of removal of di(2-ethylhexyl) phthalate for *C*_0_ equal to 10 mg/kg d.w. (at pH 3) had reached 5.2% (Figure 4a). However, where the application of hydrogen peroxide was supported by an ultrasonic field of 3.97 W/cm^2^, the degree of DEHP degradation for the lowest *C*_0_ was at 32.4%–62.3% for *t* = 5–60 min (Figure 4b). Statistically significant differences were observed for the process supported by an ultrasonic field for all initial contents.

During 5 min of exposure to the ultrasonic field under the H_2_O_2_/UD system, the value for the analysed parameter was over six times greater than with 1 h of oxidation using hydrogen peroxide alone. An increase in efficiency was also observed in the combined process, as compared with that using the ultrasonic field alone. The results of our own research on the influence of the ultrasonic field in the DEHP removal process are presented in Kida et al. [17].

#### 3.2.2. The Fenton Process as Supported by an Ultrasonic Field

**Figure 5:**
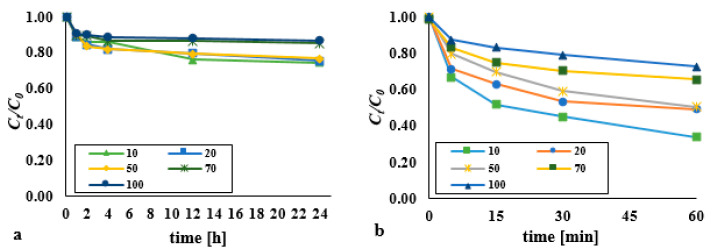
Influence of initial content on the decomposition of DEHP using (**a**) the Fenton process (pH = 3, molar ratio DEHP:H_2_O_2_:Fe^2+^ 1:1:1); (**b**) the Fenton process supported by an ultrasonic field (pH = 10, molar ratio DEHP:H_2_O_2_:Fe^2+^ 1:1:1, acoustic wave intensity of 3.97 W/cm^2^).

The effort to clean bottom sediments using Fenton’s reagent was most effective (amounting to 25.9% removal on average) where *C*_0_ for di(2-ethylhexyl) phthalate was equal to 10 mg/kg and where the reaction proceeded for 24 h (Figure 5a). There was a statistically significant difference for the DEHP content (*p* < α, *p* = 0.0171). The percentage was almost half as low for *C*_0_ = 100 mg/kg d.w., notwithstanding the removal of a total (absolute) amount of DEHP that was more than five times as great The increase in the concentration of pollutants results in an increase in the number of molecules in the solution and ensures their greater availability to HO· (to a lesser extent there is a recombination process of hydroxyl radicals). Esmaeli et al. (2011) [25] also showed a significant reduction in the degree of DEHP degradation in the Fenton process (H_2_O_2_—90 mg/L, Fe^2+^—5 mg/L) as a result of an increase in the initial concentration. Increasing the concentration of DEHP in the range from 10 to 30 mg/L resulted in a reduction of the degree of decomposition of di(2-ethylhexyl) phthalate in the aqueous solution from 90.2% to 55.8%.

5 min into the ultrasound-supported Fenton process, the removal efficiency of DEHP at *C*_0_ = 10 mg/kg d.w. was 32.9% on average. Furthermore, prolongation of the process was associated with a further increase in the parameter to 66.2% at *t* = 60 min (Figure 5b). There was a statistically significant difference for the DEHP content (*p* < α, *p* = 0.0151). No significant differences from the situation with the H_2_O_2_/UD system was to be noted. Comparison with sonification on its own, or the use of the Fenton process individually, allowed a clear synergistic effect to be inferred for all *C*_0_. For example, for *C*_0_ = 20 mg/kg d.w., the DEHP removal efficiency in the Fenton process was 11.6% for pH = 3 (*t* = 60 min), while for the ultrasound-assisted process, the efficiency increased to 50.6% (pH = 10, *t* = 60 min). The use of the ultrasonic field resulted in DEHP removal efficiency at the level of 46.3% (pH = 10, *t* = 60 min).

#### 3.2.3. A Modified Fenton Process Supported by an Ultrasonic Field

**Figure 6:**
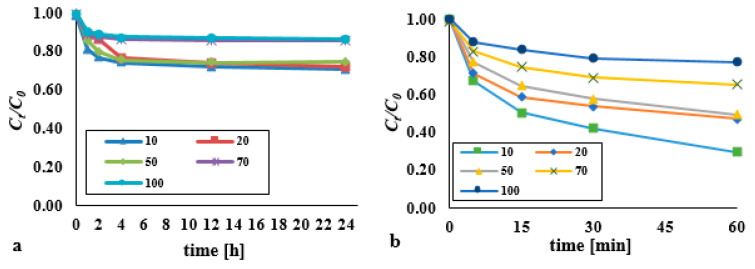
Influence of initial DEHP content on decomposition using (**a**) a modified Fenton process (pH = 3, molar ratio DEHP:H_2_O_2_:Fe^3+^ 1:1:1); (**b**) a modified Fenton process supported by an ultrasonic field (pH = 10, molar ratio DEHP:H_2_O_2_:Fe^3+^ 1:1:1, acoustic wave intensity—3.97 W/cm^2^).

Use of a modified Fenton process to clean bottom sediments of DEHP at 10 mg/kg d.w. resulted in a reduction of content by some 30% on average for *t* = 24 h. There was a statistically significant difference for the DEHP content over time (*p* < α, *p* = 0.0171). The process was characterised by higher efficiency than with the classic Fenton’s reagent. Analysis of reaction-time-dependent changes in di(2-ethylhexyl) phthalate decomposition via a modified Fenton process and *C*_0_ = 70 or 100 mg/kg d.w. showed degradation in the range 11.0%–13.7% or 8.7%–13.3% respectively (Figure 6a). These values were comparable with those for the classic Fenton reaction in the circumstances above for the initial contents of DEHP.

Where this process was supported with the acoustic wave, the highest efficiency of removal of DEHP was obtained for *C*_0_ = 10 mg/kg d.w., reaching an average value of 70.7% for *t* = 60 min (Figure 6b). There was a statistically significant difference for the DEHP content over time (*p* < *α*, *p* = 0.0027). Although the lowest efficiency of removal of DEHP applied where *C*_0_ = 100 mg/kg d.w., this was also the greatest absolute reduction of the substance from bottom sediments, due to greater accessing of the pollutants by hydroxyl radicals. The combined process was much more effective at removing DEHP from bottom sediments than the process using hydrogen peroxide, or than the classic or modified Fenton processes.

#### 3.2.4. Reaction Kinetics

**Figure 7:**
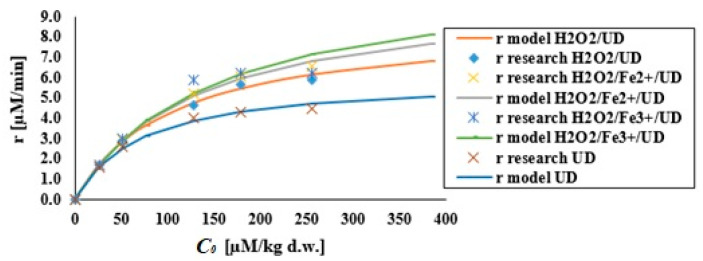
Experimental and modelled initial DEHP degradation rates for processes using the ultrasonic field (pH = 10, acoustic wave intensity—3.97 W/cm^2^), H_2_O_2_/UD (pH = 10, DEHP:H_2_O_2_ 1:1, acoustic wave intensity—3.97 W/cm^2^), H_2_O_2_/Fe^2+^/UD (pH = 10, DEHP:H_2_O_2_:Fe^2+^ 1:1:1, acoustic wave intensity—3.97 W/cm^2^), H_2_O_2_/Fe^3+^/UD (pH = 10, DEHP:H_2_O_2_:Fe^3+^ 1:1:1, acoustic wave intensity—3.97 W/cm^2^).

The kinetic description of pollutant removal usually follows a pseudo-first-order law because the degradation curve depicts an exponential decrease. However, the DEHP degradation rate increases with the initial concentration before levelling off (Figure 7). Due to the low value of Henry’s law, constant DEHP cannot be degraded by pyrolysis inside the cavitation bubble. Sono-chemical degradation of DEHP occurs in reactions at the bubble interface due to its relative high octanol/water partition coefficient. DEHP can be expected to degrade through free-radical oxidation reactions, to some extent in the bulk solution, but predominantly in the interfacial region. This suggests problems with the modelling of kinetics based solely on the pseudo-first-order constant determined at one concentration. Indeed, a linear relationship was not observed, as expected, for a first-order kinetic law. Results were presented using the initial degradation rate (μM/min). Such rates in the processes to which the ultrasonic field was applied were calculated as Δ*C*/Δ*t* in the first minutes of sonification, on the basis of results showing the change in the concentration of solute as a function of sonification time. The sono-chemical degradation reported here does not follow first-order kinetics.

For non-volatile organic compounds, the most suitable kinetic model is that developed by Okitsu et al. [30], based on organic molecules adsorbing and desorbing in respect of the liquid interface layer that surrounds the cavitation bubble, and thereby achieving a pseudo-steady state. This does not apply to substances of high volatility (HL > 10^−5^). The Okitsu et al. [30] model is described by the following equation:

(3) $$r=\frac{kKC_{0}}{1+KC_{0}}$$

where *r* is the initial degradation rate (μM/min), *C*_0_ the pollutant initial concentration, *k* the pseudo-rate constant (μM/min) and *K* the equilibrium constant (1/μM).

The model of Okitsu et al. can be linearised in five different linear forms, and the method of determining the model constants *k* and *K* for individual linear forms is as shown in Table 1. Sono-chemical degradation data for DEHP were analysed by regression analysis to fit the five linearised expressions of the model after Okitsu et al. Coefficients of determination (R^2^) showed a match between experimental and model data (Table 2), while average percentage errors (APE) were also determined after Equation (4). These indicated a match between experimental and predicted values for the initial degradation rate.

(4) $$APE\;\left(\%\right)=\frac{\sum_{i=1}^{N}\left|\frac{r_{research}-r_{model}}{r_{research}}\right|}{N}\times 100$$

N—number of experimental data

Values for the R^2^ coefficient obtained from all forms confirm that sono-chemical degradation of DEHP is consistent with the Okitsu equation. The first form—tending to be used most often—also proved most suitable in this case. Only in the hydrogen peroxide process supported by the ultrasonic field was the average percentage error slightly less favourable than that of the last form. The results differ for individual linearised forms due to changes in the structure of errors following linearisation of the non-linear equation.

To check the model from Okitsu et al. for correctness, it was necessary to recalculate initial degradation rates. The curves determined using the first form are shown in Figure 7, which offers a superposition of experimental and model results.

This model offers a near-perfect description of DEHP sono-chemical degradation data using first-form expression. It is thus appropriate to apply the linear regression coefficient in comparing best-fit equations.

### 3.3. The Impact of Acoustic-Wave Energetic Parameters on DEHP Degradation

**Figure 8:**
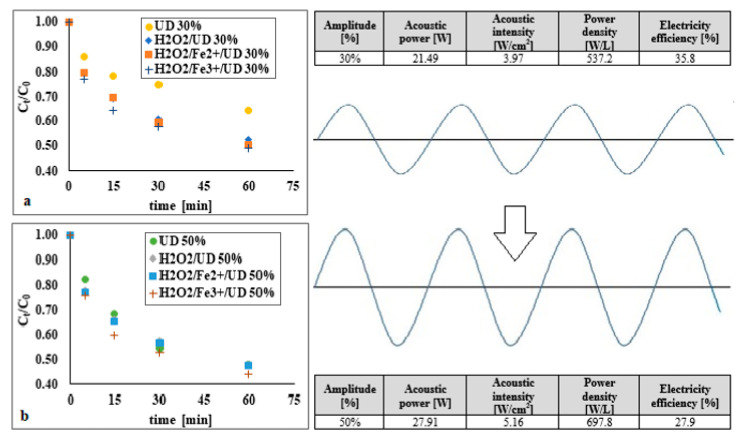
Impact of vibration amplitude on DEHP decomposition using the ultrasonic field (pH = 10), hydrogen peroxide supported by the ultrasonic field (pH = 10, DEHP:H_2_O_2_ 1:1), the Fenton process assisted by the ultrasonic field (pH = 10, molar ratio DEHP:H_2_O_2_:Fe^2+^ 1:1:1), and the modified Fenton process assisted by the ultrasonic field (pH = 10, molar ratio DEHP:H_2_O_2_:Fe^3+^ 1:1:1)-(**a**) 30%; (**b**) 50%.

The introduction of more acoustic energy into the system (in an alkaline environment) enhanced the efficiency of removal of di(2-ethylhexyl) phthalate (Figure 8). Higher vibration amplitude causes a more rapid collapse of the cavitation bubbles serving as sources of local pressure and temperature increase, and thus raising rates of reaction. In addition, increased strength of the ultrasound field from 21.49 to 27.91 W resulted in an increase in the number of cavitation bubbles, and thus amounts of hydroxyl radicals, which—due to their short lifespan—tend to merge with each other, inter alia generating more hydrogen peroxide [19,31].

The energy parameters of the ultrasonic field were determined calorimetrically [32]. Parameters for two vibration amplitudes (*A*) of 30% and 50% were analysed. The dissipated acoustic power (*P*) in the liquid was determined by measuring the rate of temperature increase due to the conversion of ultrasound energy into heat:

(5) $$P=m\cdot C_{p}\frac{\partial T}{\partial t\;}\;\left[W\right]\;$$

where: *m* is the mass of liquid (g), *C_p_* the liquid’s heat capacity (J/g∙°C) and (d*T*/d*t*) the initial slope of the curve of temperature versus time (°C/s). The values for ultrasound field energy in regard to the vibration amplitude analysed are as presented in Figure 8.

In the H_2_O_2_/UD process, a more-limited increase in DEHP degradation was observed due to increased intensity of the acoustic wave than was the case when sonification was applied in isolation. A similar circumstance also applied to the H_2_O_2_/Fe^2+^/UD and H_2_O_2_/Fe^3+^/UD systems. In the latter, the maximum differences attributable to increased vibration amplitude was of 6% on average, and 16% in the UD system. This probably reflects recombination of hydroxyl radicals in a process characterised by *A* = 50% and lower electrical efficiency.

### 3.4. The Impact of Ageing on DEHP Degradation

**Figure 9:**
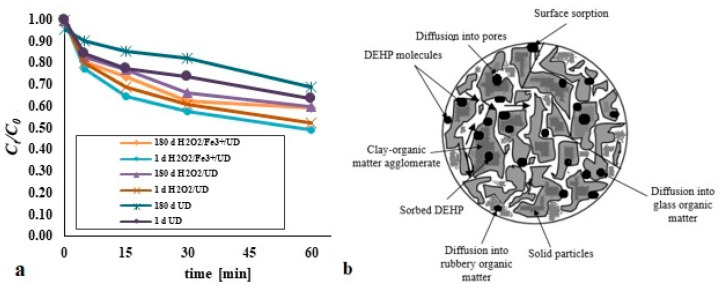
(**a**) The impact of aging contaminants on the efficiency of DEHP removal; (**b**) aging process of pollutants. Parameters: UD (*A* = 30%, pH = 10), H_2_O_2_/UD (*A* = 30%, DEHP:H_2_O_2_ 1:1, pH = 10), H_2_O_2_/Fe^2+^/UD (*A* = 30%, DEHP:H_2_O_2_:Fe^2+^ 1:1:1, pH = 10), H_2_O_2_/Fe^3+^/UD (*A* = 30%, DEHP:H_2_O_2_:Fe^3+^ 1:1:1, pH = 10).

There is wide acceptance of the idea that the sorption of pollutants by the sorbent (bottom sediment or soil) reduces the possibility of their being degraded considerably. In particular, the strong sorption properties of humic substances are such that, in combination with heavy metals, PAHs, pesticides, phthalates and other substances, they form stable complexes that are not degraded readily at all [5,33]. Tests carried out in this direction showed a slight decrease in the efficiency of removal of DEHP from bottom sediments in which the contamination had been present for a long time (*t* = 180 days) (Figure 9a). It is probable that, over time, di(2-ethylhexyl) phthalate becomes bound more and more strongly by organic matter contained in the analysed bottom sediments. This process, referred to as ageing, generally sees a given contaminant become subject to transformations yielding a more stable solid-associated compound. Slow sorption, diffusion and equilibrium division play an important role in the ageing of pollutants, the processes being referred to collectively as sequestration. Their mutual participation testifies to the scope and intensity of sequestration of the pollutants in question. It is assumed that this process in soil and bottom sediments results from diffusion into the interior of organic matter, with a solid matrix being retained inside nano- and micropores (Figure 9b) [5,33,34]. The construction of organic matter takes place via the so-called rubber and glassy phases, with both entailing active centres capable of binding organic compounds characterised by differing strengths and mechanisms of interaction with organic pollutants. In the glassy fraction, the interaction between organic matter and organic pollutants is of considerable strength. The rubber fraction has active centres interacting only weakly with hydrophobic pollutants. Organic substances associated with the organic fraction are of particularly limited susceptibility to processes of biodegradation and volatilisation, hence the high concentrations of hard-to-decompose pollutants present in bottom sediments and soil even several decades after emission or use.

Test results showed that, in the H_2_O_2_/UD process, sequestration resulted in slightly more limited DEHP degradation. Where the H_2_O_2_/Fe^3+^/UD system was applied, it was possible to achieve the highest efficiency of removal of di(2-ethylhexyl). Such a phenomenon was also presented by Sun and Yan (2008) [22], having analysed the effectiveness of removal from soil of pyrene (at 40 mg/kg d.w.) using a Fenton process (with 200 mM H_2_O_2_ and 20 mM Fe^2+^, a pH of 3 and *t* = 30 min) 30, 60 and 180 days on from the introduction of the contaminant into the matrix. The tests were carried out for soil samples of 1.6% or 5.2% organic matter content. Efficiency for *t* = 30 days compared with *t* = 1d was lower by 14.2% for OM = 1.6% and by 23.1% for OM = 5.2%. No significant differences were observed after 60 and 180 days in relation to *t* = 30 days. In the view of these authors, what happens over time is a slow transformation of absorbed particles, followed by a transformation into intro-spheric complexes.

### 3.5. Impacts on Dissolved Organic Carbon (DOC) and Organic Matter (OM)

**Figure 10:**
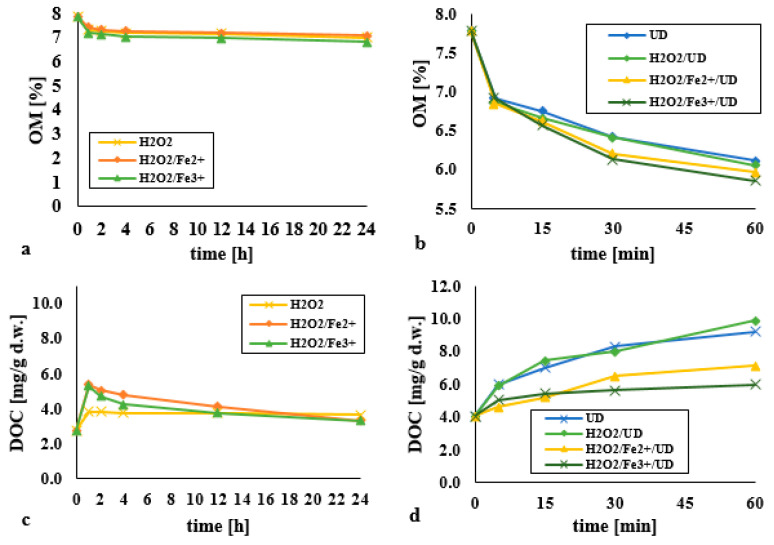
The impact on (**a**) OM exerted by the use of H_2_O_2_ (pH = 3, DEHP:H_2_O_2_ 1:1), the Fenton process (pH = 3, molar ratio DEHP:H_2_O_2_:Fe^2+^ 1:1:1), the modified Fenton process (pH = 3, DEHP:H_2_O_2_:Fe^3+^ 1:1:1) (**b**) OM exerted by the use of UD (pH = 10, acoustic wave intensity-3.97 W/cm^2^), H_2_O_2_/UD (pH = 10, DEHP:H_2_O_2_ 1:1, 3.97 W/cm^2^), H_2_O_2_/Fe^2+^/UD (pH = 10, molar ratio DEHP:H_2_O_2_:Fe^2+^ 1:1:1, 3.97 W/cm^2^), H_2_O_2_/Fe^3+^/UD (pH = 10, molar ratio DEHP:H_2_O_2_:Fe^3+^ 1:1:1, 3.97 W/cm^2^); (**c**) DOC exerted by the types of single process (**d**) DOC exerted by the type of process supported by an ultrasonic field.

Organic matter is the main component of soil capable of affecting processes associated with the transformation of persistent organic pollutants (POPs). The OM content in the analysed bottom sediments was at the level of 7.8%. This was gradually lowered as reactions proceeded (Figure 10a,b). Higher values for this parameter in processes introducing an acoustic wave may also reflect the alkaline reaction environment, which probably promotes the dissolution of OM. Incomplete decomposition of organic matter is a positive effect obtained as a result of the processes used, because OM is an important component of soil in particular. The high content of functional groups among humic substances allows for ion exchange, complex formation and adsorption with heavy metals.

The mineralisation of the organic matter in bottom sediments achieved using ultrasound increases the possibility of soluble compounds passing into the aqueous phase, as presented in the form of changes in the concentration of dissolved organic carbon (Figure 10c,d). The increase in DOC as oxidation proceeds reflects the release of lower molecular weight substances constituting the hydrophilic fraction of organic matter from the hydrophobic OM fraction. This phenomenon is probably a consequence of the HO· reaction mechanism—with electrophilic addition to alkenes or aromatic rings [35].

Reference to the OM and DOC parameters also confirms the highest efficiency of removal of DEHP associated with the H_2_O_2_/Fe^3+^/UD system. Likewise, in a modified Fenton process deployed by Cheng et al. (2016) [35], the value of DOC was four times as high (at 1.2 mg/g) when it came to the removal of atrazine (617.5 mg/kg) from soil. The authors show that oxidation with Fenton’s reagent can reduce OM content slightly, usually by about 10% compared with the initial value, as humic acids (as the main components of OM) resist oxidation. In research by Zhao et al. (2018) [32], DOC content was also four times as great as DEHP removed from bottom sediments in an H_2_O_2_/Fe^3+^ system. Content of organic matter decreased slightly from an average of 8.5% to around 7.7%.

### 3.6. The Impact of the Applied Processes on the Elution of Selected Components from the Bottom-Sediment Matrix

**Figure 11:**
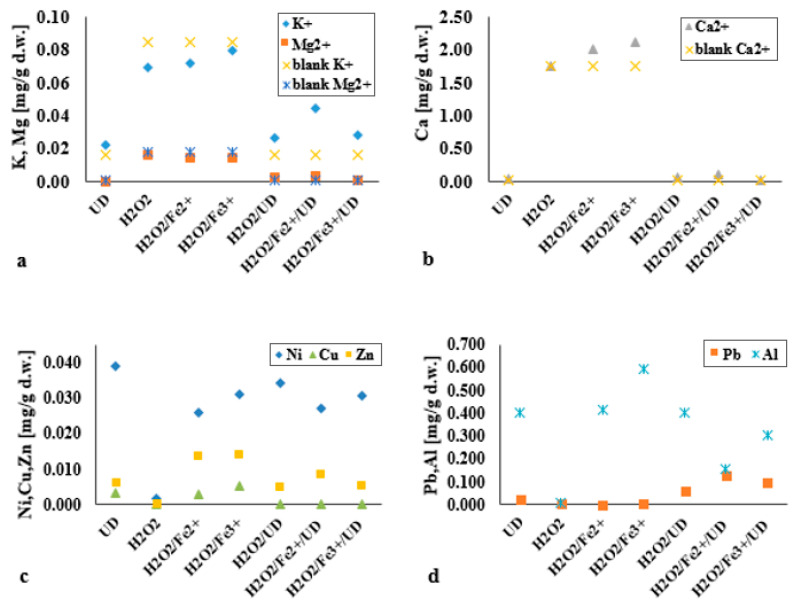
Leaching from bottom sediments of the selected macro-elements: (**a**) K^+^ and Mg^2+^ and (**b**) Ca^2+^; (**c**) Ni, Cu and Zn; and (**d**) Pb and Al. Parameters: UD (*A* = 30%, *pH* = 10, *t* = 60 min), H_2_O_2_ (DEHP:H_2_O_2_ 1:1, *pH* = 3, *t* = 60 min), H_2_O_2_/UD (*A* = 30%, DEHP:H_2_O_2_ 1:1, *pH* = 10, *t* = 60 min), H_2_O_2_/Fe^2+^ (DEHP:H_2_O_2_:Fe^2+^ 1:1:1, *pH* = 3, *t* = 60 min), H_2_O_2_/Fe^2+^/UD (*A* = 30%, DEHP:H_2_O_2_:Fe^2+^ 1:1:1, *pH* = 10, *t* = 60 min), H_2_O_2_/Fe^3+^ (DEHP:H_2_O_2_:Fe^3+^ 1:1:1, *pH* = 3, *t* = 60 min), H_2_O_2_/Fe^3+^/UD (*A* = 30%, DEHP:H_2_O_2_:Fe^3+^ 1:1:1, *pH* = 10, *t* = 60 min).

The research also pointed to leaching of bottom-sediment components such as magnesium, potassium and calcium, which are among the most important plant nutrients (Figure 11a,b). In the case of these analysed macro-elements, a clear difference in the degree of leaching was to be noted where the ultrasonic field was not applied. Respectively the most limited or greatest susceptibility to leaching characterised Mg^2+^ (in the UD system) and Ca^2+^ (in the H_2_O_2_/Fe^3+^ system). Acidification of the solid matrix enhances the leaching of nutrients from it, such that the factor determining the elution of plant nutrients was less the process involved and more the acidification of the reaction environment. This ensures that, in low-pH conditions, there is reduced absorption of minerals such as calcium, magnesium and potassium by vegetation. Equally, a change in the pH of bottom sediments to slightly acidic or acidic increases the amounts of mobile (plant-available) heavy-metal forms, given increased solubility and reduced adsorption on colloidal particles [36].

The action of ultrasound was shown to entail the release of Ni, Cu, Zn, Pb and Al from bottom sediments, even where the process was run in an alkaline reaction environment (Figure 11c,d). Physicochemical analysis in bottom sediments showed the presence of Ni, Cu, Zn, Pb, Fe and Al at the levels of 30.90 mg/kg d.w., 26.15 mg/kg d.w., 105.9 mg/kg d.w., 43.30 mg/kg d.w., 34.58 g/kg d.w., 35.15 g/kg d.w., respectively. Where the H_2_O_2_/Fe^2+^ and H_2_O_2_/Fe^3+^ systems were tested in an acidic environment, the degree of leaching of zinc from bottom sediments was enhanced above the level noted with combined processes or sonification alone. Lead and copper were released least under the H_2_O_2_/Fe^2+^ and H_2_O_2_/Fe^3+^ processes, confirming ideas in the literature that Pb is eliminated most readily at pH 2.5, and Cu at pH 3.5 [37]. No leaching of Cu was observable in the ultrasound-assisted processes. The best effects as regards the release of lead (compared with under other systems) were those obtained where processes were augmented with the acoustic wave. In this case, the factor determining the elution of Ni, Cu, Zn, Pb and Al was not the acid reaction medium. No leaching was observed in the blank samples, and the process deployed was thus responsible for the leaching of these components. The threats resulting from the increased presence of these elements in soil and bottom sediments mainly concern their leaching to surface and ground waters, ecotoxicity and phytotoxicity. The main threats to the environment and living organisms are lead, copper, zinc and nickel.

**Figure 12:**
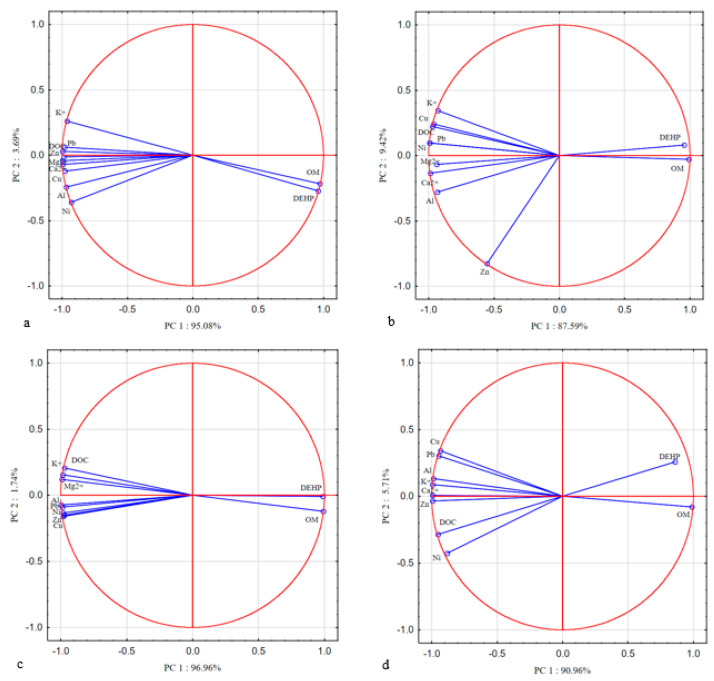
Principal Component Analysis–plot of variables. Location of load vectors towards two principal components for (**a**) UD; (**b**) H_2_O_2_/UD (**c**) H_2_O_2_/Fe^2+^/UD (**d**) H_2_O_2_/Fe^3+^/UD.

The large amount of data encouraged the use of PCA analysis to achieve a more logical grouping. This analysis will facilitate the design of processes by being able to predict the behavior/interaction of the process-relevant components. The relationships between the primary variables and the obtained principal components are presented graphically in Figure 12. PCA analysis showed a similar distribution in all processes supported by the ultrasonic field. This analysis showed that the substances analyzed for each process differentiated in each case in the characteristics associated with the one or two components. The eigenvalue for the first components for the analyzed processes ranges from 9.07 to 10.45 and the percentage of the variance explained ranges from 87.59% to 96.96%. The second component for individual processes explains much less variance in the range 1.74%–9.41% and its eigenvalues are within the range 0.17–1.04. In line with the Kaiser criterion, interpretation may be extended to those components whose eigenvalue exceeds 1. Moreover, the analysis of the landfill chart shows that the conclusion is consistent. Accordingly, the first or the first two components provide important information on the analyzed processes.

The analyzed components, apart from DEHP and OM, in each case negatively correlate with the first component. However, zinc in the H_2_O_2_/UD process is represented more by the second component. Similar relationships between the individual components were confirmed in all analyzed processes. A very high positive correlation was observed between DEHP and organic matter, and a negative relationship with other analyzed components. In turn, there was a high positive correlation between dissolved organic carbon and substances leached from the bottom sediment matrix, such as plant nutrients and selected metals. The exception is zinc in the H_2_O_2_/UD process, where a weak relationship with DOC was noted. The PCA analysis also confirms the high relationship between plant nutrients in all analyzed processes.

### 3.7. Impact of Processes Applied on the Formation of Intermediate Products

**Figure 13:**
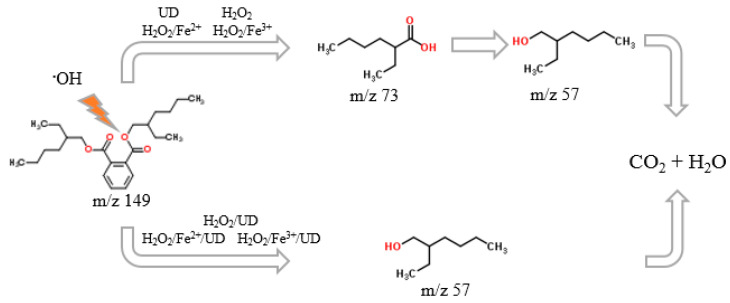
The DEHP degradation pathway.

Bottom sediments were not found to feature reaction by-products such as mono(2-ethylhexyl) phthalate (MEHP), phthalic acid or proto-catechic acid (Figure 13). Mono(2-ethylhexyl) phthalate is considered the most harmful product of the degradation of DEHP.

In aerobic conditions, the process of DEHP decomposition is associated with hydrolysis of the ester bond, with 2-ethylhexanol and mono(2-ethylhexyl) phthalate generated. In the processes analysed, these most likely transformed into phthalic acid, then into proto-catechic acid, subsequently decomposable into CO_2_ and H_2_O. However, after 60 min of the reaction, 2-ethylhexanol was not decomposed completely to 2-ethylhexanoic acid in the UD, H_2_O_2_, H_2_O_2_/Fe^2+^ and H_2_O_2_/Fe^3+^ systems. In turn, where processes were supported by the ultrasonic field, only 2-ethylhexanoic acid was a by-product.

## 4. Conclusions

- Neither a process using hydrogen peroxide, nor the classic Fenton reaction, nor a modified Fenton process proved effective enough in removing DEHP from bottom sediments. However, the deployment of an ultrasonic field in support of these processes provided for a synergistic effect on DEHP removal under optimal conditions.
- Process support for the use of hydrogen peroxide or for classic or modified Fenton processes were seen to reduce both reaction times from 24 h down to 5 min and amounts of reagents needed (for hydrogen peroxide up to 1000 times less, for iron 50 times less), in comparison with the single systems, while energy efficiency was also greater than when sonification alone was conducted.
- The energy parameters of the ultrasonic field had a significant impact on the achieved level of degradation of di(2-ethylhexyl) phthalate Increasing the vibration amplitude from 30% to 50% resulted in an increase in DEHP removal efficiency in the H_2_O_2_/UD process by 11.6% (*t* = 5 min).
- The phenomenon of the sequestration of di(2-ethylhexyl) phthalate in bottom sediments proved an important factor affecting the degree of degradation. As a result of the aging of bottom sediments in the H_2_O_2_/Fe^3+^/UD process, the DEHP removal efficiency decreased by a maximum of 9% (*t* = 60 min).
- The ultrasonic field had a beneficial effect on the decomposition of organic matter and the leaching of heavy metals from bottom sediments.
- As the introduction of the acoustic wave into the reaction system did not cause mono (2-ethylhexyl) phthalate to form, there was no intensified generation of by-products during the degradation process.
- Where the Fenton process applied to bottom sediments was supported by the ultrasonic field, there was no requirement to add catalyst, given the naturally-occurring admixture of iron in the matrix.

## Figures and Tables

**Table 1:** Investigated factors in tested processes.

Factor	H_2_O_2_	H_2_O_2_/Fe^2+^	H_2_O_2_/Fe^3+^	UD	H_2_O_2_/UD	H_2_O_2_/Fe^2+^/UD	H_2_O_2_/Fe^3+^/UD
dose	x	x	x	x	x	x	x
time	x	x	x	x	x	x	x
pH	x	x	x	x	x	x	x
initial content of DEHP	x	x	x	x	x	x	x
degradation rates	−	−	−	x	x	x	x
energetic parameters	−	−	−	x	x	x	x
ageing	−	−	−	x	x	x	x
DOC and OM	x	x	x	x	x	x	x
elution of plant nutrients	x	x	x	x	x	x	x
elution of metal	x	x	x	x	x	x	x
intermediate products	x	x	x	x	x	x	x

X: the parameter was analyzed, −: the parameter was not analyzed.

**Table 2:** Parameters for the model after Okitsu et al. obtained using the linear regression method.

Form	Parameters	UD	H_2_O_2_/UD	H_2_O_2_/Fe^2+^/UD	H_2_O_2_/Fe^3+^/UD
$\frac{1}{r}=\frac{1}{Kk}\frac{1}{C}+\frac{1}{k}$	k (µM/min)	5.96	8.70	10.20	11.14
	K (1/mM)	14.29	9.30	7.80	6.9
	R^2^	0.9973	0.9984	0.9996	0.9940
	APE (%)	2.41	2.64	1.48	5.48
$\frac{C}{r}$ $=\frac{1}{k}$ $+\frac{1}{\mathrm{Kk}}\frac{C}{r}=\frac{1}{k}C+\frac{1}{Kk}$	k (µM/min)	5.57	8.26	9.73	9.18
	K (1/mM)	16.39	10.31	8.48	9.90
	R^2^	0.9956	0.9917	0.9958	0.9536
	APE (%)	2.98	2.60	2.00	7.87
$r=-\frac{1}{K}\frac{r}{C}+k$	k (µM/min)	5.80	8.46	9.98	9.88
	K (1/mM)	15.24	9.81	8.07	8.58
	R^2^	0.9781	0.9773	0.9903	0.8345
	APE (%)	2.55	2.59	1.57	6.60
$\frac{r}{C}=-Kr+Kk$	k (µM/min)	5.85	8.55	10.03	10.88
	K (1/mM)	14.9	9.60	8.00	7.20
	R^2^	0.9781	0.9773	0.9903	0.8345
	APE (%)	2.47	2.57	1.56	5.55
$\frac{1}{C}=Kk\frac{1}{r}-K$	k (µM/min)	5.99	8.70	10.17	11.29
	K (1/mM)	14.2	9.30	7.80	6.80
	R^2^	0.9973	0.9984	0.9996	0.9940
	APE (%)	2.49	2.63	1.51	5.56

## Data Availability

The data is available on the request to the corresponding author.

## Supplementary Materials

The following are available online at https://www.mdpi.com/article/10.3390/ma14113029/s1, Table S1: World production and consumption of DEHP, Table S2: Physicochemical properties of DEHP, Table S3: Occurrence of di(2-ethylhexyl) phthalate in various environmental components, Table S4: Occurrence of di(2-ethylhexyl) phthalate in surface water, Table S5: Occurrence of di(2-ethylhexyl) phthalate in bottom sediments, Table S6: Occurrence of di(2-ethylhexyl) phthalate in soil.
