# Supplementary material for: The Use of an Ultrasonic Field in Support of Classical Methods of Oxidising Component Leached from Microplastics in Bottom Sediments
Source: Materials (Basel). 2021 Jun 2;14(11):3029. doi: 10.3390/ma14113029 (PMC8199688; doi:10.3390/ma14113029)
Supplement: Supplementary file 1 [file materials-14-03029-s001.zip › materials-1214569-supplementary.pdf]

The Use of an Ultrasonic Field in Support of Classical Methods of Oxidising Component Leached from Microplastics in Bottom Sediments

Małgorzata Kida[1], Sabina Ziembowicz, Piotr Koszelnik

Table S1. World production and consumption of DEHP

Location	Year	Production/use thousand tons/year	Literature
**EU**	–	500	Magdouli et al., 2013
**Western Europe**	1997	595	Roslev et al., 2007
	1999	476	
	2004	221	
**Canada**	1991	10	Minister of Supply and Services Canada, 1994
**Japan**	–	204	Magnusalnervik et al., 1997
**Germany**	–	144	Magnusalnervik et al., 1997
**USA**	2006	45-230	Tecnon Orbichem, 2007
**China**	2006–2007	305-340	Tecnon Orbichem, 2007

Table S2. Physicochemical properties of DEHP [Peijnenburg and Struijs, 2006; Net et al., 2015]

Physicochemical properties	*Unit*	*DEHP*	*Structural formula*
Chemical formula		$C_{24}H_{38}O_4$	
Molecular weight	g/mol	390.54	
Vapor pressure at 200°C	hPa	1.6	
Vapor pressure at temperature 20°C	Pa	$3.4·10^{-5}$	
Water solubility	mg/dm³	$2.49·10^{-3}$	
Color		colorless	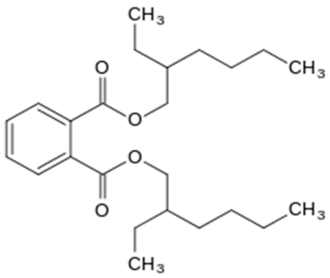
Physical state at temperature 20°C		oily liquid	
Log K_{OW}		7.73	
Log K_{OA}		10.53	
Log K_{AW}		–2.8	
K_{OC}	dm³/kg	$87\,420–51·10^4$	
pKa (strongly alkaline environment)		–6.7	
H	Pa·m³/mol	4.43	
Density	g/cm³	0.98	
Boiling point	°C	385	
Melting temperature	°C	–55	
Flash-point	°C	193	
Temperature of self-ignition	°C	400	

K_{OA}–octanol-air partition, K_{OW}–octanol–water partitioning, K_{AW}–air–water partitioning, K_{OC}–organic carbon partitioning, pK_a–dissociation constant, H–Henry's constant.

[1] Corresponding author. Email: mkida@prz.edu.pl

Table S3. Occurrence of di(2-ethylhexyl) phthalate in various environmental components

Matrix	Type	Location	DEHP [ng/dm³]				Literature
			min	*max*	*average*	*median*	
Drinking water	tap water	Spain	-	-	445	-	Blanchard et al., 2013
		China	-	-	12, 487	-	Tang et al., 2012
		France	-	-	350	-	Blanchard et al., 2013
	bottled water	Spain	ND	1520	-	-	Guart et al., 2014
		Hungary	16	1700	-	-	Keresztes et al., 2013
		Canada	360	460	-	-	Cao et al., 2008
		Egypt	ND	298	104	67	Zaki et al.,
Underground water		Spain	-	5661	-	-	López-Roldán et al., 2004
Rain		Netherland s	0.69	1.7	-	-	Vethaak et al.., 2005
Air		Shanghai, China	0.150	0.368	-	-	Wang et al., 2006
		Zhejiang, China	0.097	0.301	-	-	Wang et al., 2006
		Guangdon g, China	0.112	0.250	-	-	Wang et al., 2006

ND – no data

Table S4. Occurrence of di(2-ethylhexyl) phthalate in surface water

Type	Location	DEHP [μg/dm³]				Literature
		min	*max*	*average*	*median*	
**River**	Veldwachters, South Africa	80	431	-	-	Olujimi et al., 2012
	Klang, Malaysia	3.1	64.3	-	-	Tan, 1995
	Yangtze River, China	12.07	2127	-	-	Zhang et al., 2018
	Rhône, France	0.04	0.407	-	-	Paluselli et al., 2017
	Somme, France	5.16	20.76	10.23	8.88	Net et al., 2014
	Jiulong, China	0.62	12.43	-	-	Li et al., 2017
	Yangtze River, China	0.01	54.7	-	-	Wang et al., 2008a
	Dam, Japan	ND	3.60	-	-	Suzuki et al., 2001

	Dommel, the Netherlands	0.9	5	-	3.2	Vethaak et al., 2005
	Kaweri, India	ND	0.822	-	-	Selvaraj et al., 2015
	Ebro, Muga, Fluvia, Ter, Besos, Llobregat, Spain	0.12	4.98	1.19	-	Sanchez-Avila et al., 2012
	Ebro, Spain	0.136	32.86	2.28	-	Catalan Water Agency, 2011
	Wangyang, China	0.26	0.94	0.48	0.43	Zhang et al., 2015
	Rivers of the Eastern Cape, South Africa	0.06	2306	-	-	Fatoki andNoma, 2002
	Rhine, Germany	25	36	-	-	Fromme et al., 2002
	Supraśl, Polska	-	8.67	1.04	-	GIOŚ, 2016
	Horodnianka, Poland	-	1.63	0.81	-	GIOŚ, 2016
		-	-	1.78	-	GIOŚ, 2016
	Czarna, Poland	-	0.66	0.24	-	GIOŚ, 2016
	Liza, Poland	-	0.98	0.23	-	GIOŚ, 2016
	Mała Panew, Poland	-	0.40	-	-	GIOŚ, 2016
	Łukawica, Poland	-	-	0.3	-	GIOŚ, 2016
	Biała, Poland	-	-	3.22	-	GIOŚ, 2016
**Lake**	15 lakes in the city of Guangzhou, China	0.237	1.60	-	-	Zeng et al., 2008a
	Tuczno, Poland	-	-	0.29	-	GIOŚ, 2013
		-	-	0.65	-	GIOŚ, 2014
	Kłeckie, Poland	-	-	0.65	-	GIOŚ, 2014
	Skąpe, Poland	-	-	0.405	-	GIOŚ, 2014

ND – no data

Table S5. Occurrence of di(2-ethylhexyl) phthalate in bottom sediments

Type	Location	DEHP [mg/kg d.w.]				Literature
		min	*max*	*average*	*median*	
**River**	Meuse, Rhine, Scheldt, the Netherlands	0.123	7.6	0.6	-	Vethaak et al., 2005
	Wangyang, China	0.161	0.465	0.307	0.304	Zhang et al., 2015
	Yellow River, China	6.35	259	-	45.7	Sha et al., 2007
	Klang, Malaysia	0.49	15.0	-	1.63	Tan, 1995
	Houjing, Taiwan	0.10	20.22	7.0	-	Lin et al., 2009
	Yangtze River, China	13.9	322	-	144	Wang et al., 2008b
	Pearl River, China	0.415	29.5	-	1.46	Liu et al., 2014
	Ogun, Nigeria	0.02	0.82	0.172	0.05	Adeniyi et al., 2011
	Trent, UK	0.84	31.0	-	-	Long et al., 1998
	Aire, UK	7.89	115.2	26.63	-	Long et al., 1998

	Love, Salt, Canon, Taiwan	0.40	34.8	5.02	-	Chen et al., 2013
	Jiulong, China	0.053	1.28	0.35	0.19	Li et al., 2017
	Odra, Poland	-	-	1.19	-	GIOŚ, 2016
	Parsęta, Poland	-	-	1.94	-	GIOŚ, 2016
**Lake**	Wu, China	-	-	202.3	-	Wang et al., 2008b
	East, China	-	-	323.5	-	Wang et al., 2008b
	Wielkie Dąbie, Poland	-	-	4.17	-	GIOŚ, 2016
				<0.1		GIOŚ, 2013
	Lubinieckie, Poland	-	-	1.20	-	GIOŚ, 2015

GIOŚ - Chief Inspectorate for Environmental Protection in Poland

Table S6. Occurrence of di (2-ethylhexyl) phthalate in soil

**Type**	**Location**	**DEHP [mg/kg d.w.]**				**Literature**
		min	*max*	*average*	*median*	
**Area agricultural**	Guangzhou, China	0.180	29.37	4.09	0.308	Zeng et al., 2008b
	Spain	1	63	-	-	Plaza-Bolanos et al., 2012
	Shijiazhuang, China	0.066	0.263	0.143	0.132	Zhang et al., 2015
	Nanjing, China	0.240	4.18	0.184	0.194	Wang et al., 2015
	Yangtze River Delta, China	ND	9.19	0.546	0.349	Sun et al., 2013
	Amursko-Sungarian Plain, China	0.077	0.583	0.222	0.203	Wang et al., 2017
	Xinjiang, China	104	149	128.7	-	Guo and Wu, 2011
	France	-	-	0.242	-	Tran et al., 2015
	Czech Republic	0.16	1.02	-	-	Dankova et al., 2012
	Roskilde, Denmark	0.012	1.9	-	-	Vikelsøe et al., 2002
	UK	0.022	0.075	-	-	Gibson et al., 2005
	China	ND	6.22	0.821	0.562	Niu et al., 2014
**Urban area**	Guangzhou, China	1.41	264	63	31.9	Zeng et al., 2009
	Tianjin, China	0.028	4.17	0.618	0.099	Kong et al., 2012
	Scotland, Great Britain	0.025	1.596	0.162	0.110	Rhind et al., 2013
**Recreation area**	Novi Sad, Serbia	0.164	2.04	0.844	-	Škrbić et al., 2016
	Tianjin, China	0.043	0.801	0.179	0.125	Zhao et al., 2018
	Guangzhou, China	0.892	154	29.4	10.4	Zeng et al., 2009

ND–no data

Cavitation bubbles appear as a result of local disruption of the medium under the influence of high tensile forces. During the operation of the ultrasonic field, cavitation bubbles increase their radius twice or more. Subsequently, they collapse rapidly, resulting in the release of a large amount of energy, resulting in a local increase in temperature and pressure. A sharp increase in pressure inside the bubble causes them to crack and the formation of a hydrodynamic shock wave. Extreme conditions are the reason for the thermal breakdown of water molecules, during which hydroxyl radicals are formed, which are the main source of so-called sonochemical reactions (reactions 1–13). This phenomenon significantly accelerates the course of chemical and physical reactions [Miłowska, 2007; Bogacki and Naumczyk, 2009].

$$H_2O +))) \longrightarrow HO\cdot + H\cdot \quad (1)$$

$$O_2 +))) \longrightarrow 2O\cdot \quad (2)$$

$$HO\cdot + O\cdot \longrightarrow HOO\cdot \quad (3)$$

$$O\cdot + H_2O\cdot \longrightarrow 2HO\cdot \quad (4)$$

$$H\cdot + O_2\cdot \longrightarrow HOO\cdot \quad (5)$$

$$OH\cdot + H\cdot \longrightarrow H_2O\cdot \quad (6)$$

$$2HO\cdot \longrightarrow H_2O\cdot + O\cdot \quad (7)$$

$$HOO\cdot + HO\cdot \longrightarrow O_2 + H_2O \quad (8)$$

$$2HO\cdot \longrightarrow H_2O_2 \quad (9)$$

$$2HOO\cdot \longrightarrow H_2O_2 + O_2 \quad (10)$$

$$H\cdot + H_2O_2 \longrightarrow HO\cdot + H_2O \quad (11)$$

$$HO\cdot + H_2O_2 \longrightarrow HOO\cdot + H_2O \quad (12)$$

$$2H\cdot \longrightarrow H_2 \quad (13)$$

Fenton's reaction is a complex process that involves many side reactions. In soil, bottom sediments, these reactions include basic radical initiation (reactions (14) and (15)), propagation (reactions (16) and (17)) and termination (reactions (18)–(20)), as well as reactions with intermediates (reactions (21)–(28)), with carbonate and bicarbonate ions (reactions (29)–(34)) and with natural organic matter (NOM) (reactions (35)) [Yap et al., 2011 ; Gan and Ng, 2012; Cheng et al., 2016].

$$H_2O_2 + Fe^{2+} \longrightarrow Fe^{3+} + HO\cdot + OH^- \quad (14)$$

$$Fe^{3+} + H_2O_2 \longrightarrow Fe^{2+} + HO_2\cdot + H^+ \quad (15)$$

$$HO\cdot + RH \longrightarrow R\cdot + H_2O \quad (16)$$

$$R\cdot + H_2O_2 \longrightarrow ROH + HO\cdot \quad (17)$$

$$HO\cdot + Fe^{2+} \longrightarrow Fe^{3+} + OH^- \quad (18)$$

$$HO\cdot + H_2O_2 \longrightarrow H_2O + HO_2\cdot \quad (19)$$

$$HO\cdot + HO\cdot \longrightarrow H_2O_2 \quad (20)$$

$$Fe^{2+} + HO_2\cdot \longrightarrow Fe^{3+} + HO_2^- \quad (21)$$

$$Fe^{3+} + HO_2\cdot \longrightarrow Fe^{2+} + O_2 + 2H^+ \quad (22)$$

$$HO_2\cdot + H_2O_2 \longrightarrow H_2O + OH\cdot + O_2 \quad (23)$$

$$HO_2\cdot + HO_2\cdot \longrightarrow H_2O + O_2 \quad (24)$$

$$O_2\cdot^- + Fe^{3+} \longrightarrow Fe^{2+} + O_2 \quad (25)$$

$$O_2\cdot^- + Fe^{2+} + 2H^+ \longrightarrow Fe^{3+} + H_2O_2 \quad (26)$$

$$HO\cdot_2 + O_2\cdot^- + H_2O \longrightarrow H_2O_2 + O_2 + OH^- \quad (27)$$

$$HO\cdot + HO_2\cdot \longrightarrow H_2O + O_2 \quad (28)$$

$$CO_2(g) + H_2O \longleftrightarrow H_2CO_3(aq) \quad (29)$$

$$H_2CO_3(aq) \longleftrightarrow HCO_3^- + H^+ \quad (30)$$

$$HCO_3^- + OH\cdot \longrightarrow CO_3^- + OH^- \quad (31)$$

$$CO_3^- + H_2O_2 \longrightarrow HO_2\cdot + HCO_3^- \quad (32)$$

$$2CO_3^- \longrightarrow \text{products} \quad (33)$$

$$CaCO_3(s) + H^+ \longleftrightarrow Ca^{2+} + HCO_3^- \quad (34)$$

$$NOM + HO\cdot \longrightarrow \text{products} \quad (35)$$

References

1. Adeniyi A. A., Okedeyi O. O., Yusuf K. A. Flame ionization gas chromatographic determination of phthalate esters in water, surface sediments and fish species in the Ogun river catchments, Ketu, Lagos, Nigeria. *Environmental monitoring and assessment* **172(1–4)**, 561–569 (2011).
2. Blanchard M., Teil M. J., Dargnat C., Alliot F., Chevreuil M. Assessment of adult human exposure to phthalate esters in the urban centre of Paris (France). *Bulletin of environmental contamination and toxicology* **90(1)**, 91–6 (2013).
3. Bogacki J., Naumczyk J. Ex-situ physicochemical methods for the treatment of heavy metal bottom sediments. *Gaz, Woda i Technika Sanitarna* **11**, 3–42 (2009).
4. Cao X. L. Determination of phthalates and adipate in bottled water by headspace solid – phase microextraction and gas chromatography/mass spectrometry. *Journal of Chromatography A*, **1178(1–2**), 231–238 (2008).
5. Catalan Water Agency. Historical data of pesticides and organic persistent compounds in Catalan coastal waters for the period 2007–2010. Resultats organics Maresme Ebre 2007 – 2010 (2011).
6. Chen C. W., Chen C. F., Dong C. D. Distribution of phthalate esters in sediments of Kaohsiung Harbor, Taiwan. *Soil and Sediment Contamination: An International Journal* **22(2)**, 119–131 (2013).
7. Cheng M., Zeng G., Huang D., Lai C., Xu P., Zhang C., Liu Y. Hydroxyl radicals based advanced oxidation processes (AOPs) for remediation of soils contaminated with organic compounds: a review. *Chemical Engineering Journal* **284**, 582–598 (2016)
8. Dankova R., Jarošova A., Polakova Š. The monitoring of dibutyl phthalate and di–2–ethylhexyl phthalate in Moravian agricultural soils. *Mendel Net* **35**, 736–747 (2012).
9. Fatoki O.S. Noma A. Solid phase extraction method for selective determination of phthalate esters in the Aquatic Environment. *Water Air Soil Pollut*. **140**, 85–98 (2002).

10. Fromme H., Küchler T., Otto T., Pilz K., Müller J., Wenzel A. Occurrence of phthalates and bisphenol A and F in the environment. *Water research* **36**(6), 1429–1438 (2002).

11. Gan S., Ng H. K. Modified Fenton oxidation of polycyclic aromatic hydrocarbon (PAH)-contaminated soils and the potential of bioremediation as post–treatment. *Science of the Total Environment* **419**, 240–249 (2012a).

12. Gibson R., Wang M. J., Padgett E., Beck A. J. Analysis of 4-nonylphenols, phthalates, and polychlorinated biphenyls in soils and biosolids. *Chemosphere*, **61**(9), 1336–1344 (2005).

13. GIOŚ Chief Inspectorate for Environmental Protection. Website: http://www.gios.gov.pl/pl/stan-srodowiska/monitoring-wod, access: 06.04.20 (2013–2016).

14. Guart A., Bono–Blay F., Borrell A., Lacorte S. Effect of bottling and storage on the migration of plastic constituents in Spanish bottled waters. *Food chemistry* **156**, 73– 80 (2014).

15. Guo D. M., Wu Y. Determination of phthalic acid esters of soil in south of Xinjiang cotton fields. *Arid environmental monitoring* **25**(2), 76–79 (2011).

16. Keresztes S., Tatár E., Czégény Z., Záray G., Mihucz V. G. Study on the leaching of phthalates from polyethylene terephthalate bottles into mineral water. *Science of the total environment*, 458, 451–458 (2013).

17. Kong S., Ji Y., Liu L., Chen L., Zhao X., Wang J., ... & Sun Z. Diversities of phthalate esters in suburban agricultural soils and wasteland soil appeared with urbanization in China. *Environmental Pollution* **170**, 161–168 (2012).

18. Li R., Liang J., Gong Z., Zhang N., Duan H. Occurrence, spatial distribution, historical trend and ecological risk of phthalate esters in the Jiulong River, Southeast China. *Science of the Total Environment* **580**, 388–397 (2017).

19. Lin C., Lee C. J., Mao W. M., Nadim F. Identifying the potential sources of di-(2-ethylhexyl) phthalate contamination in the sediment of the Houjing River in southern Taiwan. *Journal of hazardous materials* **161**(1), 270–275 (2009).

20. Liu H., Cui K., Zeng F., Chen L., Cheng Y., Li, H., ... & Luan T. Occurrence and distribution of phthalate esters in riverine sediments from the Pearl River Delta region, South China. *Marine pollution bulletin* **83**(1), 358–365 (2014).

21. Long J. L., House W. A., Parker A., Rae J. E. Micro – organic compounds associated with sediments in the Humber rivers. *Science of the Total Environment* **210**, 229–253 (1998).

22. López–Roldán P., López de Alda M. J., Barceló D. Simultaneous determination of selected endocrine disrupters (pesticides, phenols and phthalates) in water by in –field solid – phase extraction (SPE) using the prototype PROFEXS followed by on – line SPE (PROSPEKT) and analysis by liquid chromatography-atmospheric pressure chemicalionisation – mass spectrometry. *Anal. Bioanal. Chem.* **378**, 599–609 (2004).

23. Magdouli S., Daghrir R., Brar S. K., Drogui P., Tyagi R. D. Di 2–ethylhexylphtalate in the aquatic and terrestrial environment: A critical review. *Journal of Environmental Managemen*t **127**, 36–49 (2013).

24. Magnusalnervik J., Nejonsson S., Svensoon B. Influence of water solubility, side – chain degradability, and side – chain structure on the degradation of phthalic acid esters under methanogenic conditions. *Environ. Sci. Technol.* **31**, 2761–2764 (1997).

25. Miłowska K. Ultrasound–mechanisms of action and application in sonodynamic therapy. Postepy Hig Med Dosw.(online) **61**, 338–349 (2007).

26. Minister of Supply and Services Canada Catalogue No. En 40e215/37E, ISBN 0–662–22031–5, *Canadian Environmental Protection Act.* (1994).

27. Net S., Delmont A., Sempéré R., Paluselli A., Ouddane B. Reliable quantification of phthalates in environmental matrices (air, water, sludge, sediment and soil): A review. *Science of the Total Environment* **515**, 162–180 (2015).

28. Net S., Dumoulin D., El–Osmani R., Rabodonirina S., Ouddane B. Case study of PAHs, Me–PAHs, PCBs, phthalates and pesticides contamination in the Somme River water, France. *International Journal of Environmental Research* **8**(4), 1159–1170 (2014).
29. Niu L., Xu Y., Xu C., Yun L., Liu W. Status of phthalate esters contamination in agricultural soils across China and associated health risks. *Environmental pollution* **195**, 16–23 (2014).
30. Olujimi O. O., Fatoki O. S., Odendaal J. P., Daso A. P. Chemical monitoring and temporal variation in levels of endocrine disrupting chemicals (priority phenols and phthalate esters) from selected wastewater treatment plant and freshwater systems in Republic of South Africa. *Microchemical Journal* **101**, 11–23 (2012).
31. Paluselli A., Aminot Y., Galgani F., Net S., Sempéré R. Occurrence of phthalate acid esters (PAEs) in the northwestern Mediterranean Sea and the Rhone River. *Progress in Oceanography* (2017).
32. Peijnenburg W. J., Struijs J. Occurrence of phthalate esters in the environment of the Netherlands. *Ecotoxicology and environmental safety* **63**(2), 204–215 (2006).
33. Plaza–Bolanos P., Padilla–Sanchez J.A., Garrido–Frenich A., Romero–Gonzalez R., Martinez–Vidal J.L. Evaluation of soil contamination in intensive agricultural areas by pesticides and organic pollutants: south–eastern Spain as a case study. *J. Environ. Monit.* **14**(4), 1182–1189 (2012).
34. Rhind S. M., Kyle C. E., Kerr C., Osprey M., Zhang Z. L., Duff E. I., ... & Bell J. Concentrations and geographic distribution of selected organic pollutants in Scottish surface soils. *Environmental pollution* **182**, 15–27 (2013).
35. Roslev P., Vorkamp K., Aarup J., Frederiksen K., Nielsen P. H. Degradation of phthalate esters in an activated sludge wastewater treatment plant. *Water research* **41**(5), 969–976 (2007).
36. Sanchez–Avila J., Tauler R., Lacorte S. Organic micropollutants in coastal waters from NW Mediterranean Sea: sources distribution and potential risk. *Environ. Int.* **46**, 50–62 (2012).
37. Selvaraj K. K., Sundaramoorthy G., Ravichandran P. K., Girijan G. K., Sampath S., Ramaswamy B. R. Phthalate esters in water and sediments of the Kaveri River, India: environmental levels and ecotoxicological evaluations. *Environmental geochemistry and health* **37**(1), 83–96 (2015).
38. Sha Y., Xia X., Yang Z., Huang G. H. Distribution of PAEs in the middle and lower reaches of the Yellow River, China. *Environmental monitoring and assessment* **124**(1 – 3), 277–287 (2007).
39. Škrbić B.D., Ji Y., Đurišić-Mladenović N., Zhao J. Occurence of the phthalate esters in soil and street dust samples from the Novi Sad city area, Serbia, and the influence on the children's and adults' exposure. *Journal of Hazardous Materia*ls (2016).
40. Sun J., Huang J., Zhang A., Liu W., Cheng W. Occurrence of phthalate esters in sediments in Qiantang River, China and inference with urbanization and river flow regime. *Journal of Hazardous Materials* **248**, 142–149 (2013).
41. Suzuki T., Yaguchi K., Suzuki S., Suga T. Monitoring of phthalic acid monoesters in river water by solid–phase extraction and GC–MS determination. *Environmental science & technology* **35**(18), 3757–3763 (2001).
42. Tan G. H. Residue levels of phthalate esters in water and sediment samples from the Klang River basin. *Bulletin of Environmental Contamination and Toxicology* **54**(2), 171–176 (1995).
43. Tang C. Y., Li A. Q., Guan Y. B., Yan, L. I., Cheng X. M., Ping L. I., ... & Cui L. X. Influence of polluted SY River on child growth and sex hormones. *Biomedical and Environmental Sciences* **25**(3), 291–296 (2012).
44. Tecnon Orbichem Chemical Business Focus e a Monthly Roundup and Analysis of the Key Factors Shaping World Chemical Markets **314** (2007).

45. Tran B. C., Blanchard M., Alliot F., Chevreuil M. Fate of phthalates and BPA in agricultural and non-agricultural soils of the Paris area (France). *Environmental Science and Pollution Research* **22**(14), 11118–11126 (2015).
46. Vethaak A. D., Lahr J., Schrap S. M., Belfroid A. C., Rijs G. B. J., Gerritsen A., de Boer J., Bulder A. S., Grinwis G. C. M., Kuiper R. V., Legler J., Murk T. A. J., Peijnenburg W., Verhaar H. J. M., de Voogt P. An integrated assessment of estrogenic contamination and biological effects in the aquatic environment of The Netherlands. *Chemosphere* **59**, 511–524 (2005).
47. Vikelsøe J., Thomsen M., Carlsen L. (2002). Phthalates and nonylphenols in profiles of differently dressed soils. *Science of the Total Environment* **296**(1–3), 105–116.
48. Wang F., Sha Y. J., Xia X. H., Liu H. Distribution characteristics of phthalic acid esters in the Wuhan section of the Yangtze River. *Huan jing ke xue= Huanjing kexue* **29**(5), 1163–1169 (2008a).
49. Wang F., Xia X., Sha Y. Distribution of phthalic acid esters in Wuhan section of the Yangtze River, China. *Journal of Hazardous Materials* **154**(1–3), 317–324 (2008b).
50. Wang G., Kawamura K., Lee S., Ho K., Cao J. Molecular, seasonal, and spatial distributions of organic aerosols from fourteen Chinese cities. *Environmental Science & Technology* **40**(15), 4619–4625 (2006).
51. Wang H., Liang H., Gao D. W. Occurrence and risk assessment of phthalate esters (PAEs) in agricultural soils of the Sanjiang Plain, northeast China. *Environmental Science and Pollution Research* **24**(24), 19723–19732 (2017).
52. Wang J., Chen G., Christie P., Zhang M., Luo Y., Teng Y. Occurrence and risk assessment of phthalate esters (PAEs) in vegetables and soils of suburban plastic film greenhouses. *Science of the Total Environment* **523**, 129–137 (2015).
53. Yap C. L., Gan S., Ng H. K. Fenton based remediation of polycyclic aromatic hydrocarbons-contaminated soils. *Chemosphere* **83**(11), 1414–1430 (2011).
54. Zaki G., Shoeib T. Concentrations of several phthalates contaminants in Egyptian bottled water: Effects of storage conditions and estimate of human exposure. *Science of The Total Environment* **618**, 142–150 (2018).
55. Zeng F., Cui K., Xie Z., Liu M., Li Y., Lin Y., ... & Li F. Occurrence of phthalate esters in water and sediment of urban lakes in a subtropical city, Guangzhou, South China. *Environment International* **34**(3), 372–380 (2008).
56. Zeng F., Cui K., Xie Z., Wu L., Liu M., Sun G., ... & Zeng Z. Phthalate esters (PAEs): emerging organic contaminants in agricultural soils in peri–urban areas around Guangzhou, China. *Environmental Pollution* ***156****(2), 425–434 (2008b).*
57. Zeng F., Cui K., Xie Z., Wu L., Luo D., Chen L., ... & Sun G. Distribution of phthalate esters in urban soils of subtropical city, Guangzhou, China. *Journal of Hazardous Materials* **164**(2–3), 1171–1178.
58. Zhang Y., Liang Q., Gao R., Hou H., Tan W., He X., ... & Wang X. (2015). Contamination of phthalate esters (PAEs) in typical wastewater-irrigated agricultural soils in Hebei, North China. *PloS one* **10**(9), 0137998 (2009).
59. Zhang Z. M., Zhang H. H., Zhang J., Wang Q. W., Yang G. P. Occurrence, distribution, and ecological risks of phthalate esters in the seawater and sediment of Changjiang River Estuary and its adjacent area. *Science of The Total Environment* **619**, 93-102 (2018).
60. Zhao G., Sheng Y., Wang C., Yang J., Wang Q., Chen L. In situ microbial remediation of crude oil-soaked marine sediments using zeolite carrier with a polymer coating. *Marine pollution bulletin* **129**(1), 172–178 (2018).
